# Spatially Compartmentalized Electrospun/Electrosprayed PHB/PEO/Zein Fibrous Platforms for Dual Delivery of Rutin and *Melissa officinalis* Extract

**DOI:** 10.3390/polym18141774

**Published:** 2026-07-20

**Authors:** Dilyana Paneva, Selin Kyuchyuk, Milena Ignatova, Nevena Manolova, Iliya Rashkov, Ani Georgieva, Reneta Toshkova, Mariana Kamenova-Nacheva

**Affiliations:** 1 Laboratory of Bioactive Polymers, Institute of Polymers, Bulgarian Academy of Sciences, Akad. G. Bonchev St, bl. 103A, 1113 Sofia, Bulgaria; panevad@polymer.bas.bg (D.P.); selin.erdinch@polymer.bas.bg (S.K.); manolova@polymer.bas.bg (N.M.); rashkov@polymer.bas.bg (I.R.); 2 Centre of Competence “Sustainable Utilization of Bio-Resources and Waste of Medicinal and Aromatic Plants for Innovative Bioactive Products” (BIORESOURCES BG), 1000 Sofia, Bulgaria; 3 Institute of Experimental Morphology, Pathology and Anthropology with Museum, Bulgarian Academy of Sciences, Akad. G. Bonchev St, bl. 25, 1113 Sofia, Bulgaria; georgieva_any@abv.bg (A.G.); rtoshkova@bas.bg (R.T.); 4 Institute of Organic Chemistry with Centre of Phytochemistry, Bulgarian Academy of Sciences, Akad. G. Bonchev St., bl. 9, 1113 Sofia, Bulgaria; mariana.nacheva@orgchm.bas.bg; 5 Research and Development and Innovation Consortium, Sofia Tech Park JSC, 111 Tsarigradsko Shose Blvd., 1784 Sofia, Bulgaria

**Keywords:** poly(3-hydroxybutyrate), poly(ethylene oxide), zein, rutin, *Melissa officinalis* extract, electrospinning, electrospraying, antioxidant activity, anticancer efficacy

## Abstract

Spatially separated dual-bioactive delivery platform from electrospun polyhydroxybutyrate/poly(ethylene oxide) fibers loaded with rutin (PHB/PEO/RUT) and decorated with zein/*Melissa officinalis* particles (zein/MO) were obtained by simultaneous electrospinning/electrospraying. The morphology of the materials, their thermal properties and chemical composition were systematically studied by scanning electron microscopy (SEM), differential scanning calorimetry (DSC), thermogravimetric analysis (TGA), and attenuated total reflectance Fourier transform infrared spectroscopy (ATR–FTIR). The incorporation of PEO into the fabricated fibrous materials enhanced their wettability. It was demonstrated that the newly developed materials had high encapsulation efficiency (99.8 ± 0.1%) of RUT and/or MO. The architecture of the materials affected the in vitro release profile of the bioactive agents. RUT exerted its DPPH^•^ scavenging capacity upon incorporation into the fibers. An increase in antioxidant activity was observed in the fibrous mats loaded with both RUT and MO. Moreover, the developed materials decreased the viability of SH-4 melanoma cells to a greater extent than that of non-cancerous HaCaT keratinocytes. The combined rapid release and sustained release of bioactive agents and the antioxidant and anticancer activity of the newly developed materials render them promising candidates as platforms for local drug delivery.

## 1. Introduction

The electrohydrodynamic processes electrospinning and electrospraying have emerged as highly versatile techniques for the design of innovative and efficient micro- and nanoscale carriers of natural or synthetic bioactive agents [1,2,3,4,5,6]. Both methods are based on the application of a high voltage to a polymer-based solution, the main difference between them being the polymer concentration [7,8]. In electrospinning, the polymer concentration in the solutions is higher than that required to ensure a sufficient number of polymer chain entanglements, enabling the formation of a continuous jet and, consequently, continuous fibers. For electrospraying purposes, the polymer concentration in the solutions is lower than that required to achieve a sufficient number of chain entanglements. As a result, the application of a high voltage leads to the formation of micro- or nanosized polymer particles. The simultaneous application of the both electrohydrodynamic processes enables the preparation of composite fibrous materials that combine polymer-based nanofibers and particles possessing different functionalities [9,10]. The functionality of these materials can be further enriched by the targeted incorporation of selected low-molecular-weight bioactive agents into either the fibers and/or particles [10,11,12,13,14]. This can be easily achieved by incorporating the active compounds into the polymer solution used for electrospinning and/or electrospraying. Such approach enables tuning of the release profiles of the incorporated bioactive compounds, thereby enhancing their therapeutic efficacy while reducing potential side effects.

Poly(3-hydroxybutyrate) (PHB), a representative of the aliphatic polyesters, is a suitable candidate for the fabrication of fibrous materials by electrospinning or electrospinning/electrospraying. This is attributed to its bio-derived origin, biodegradability, and biocompatibility [15,16,17]. The properties of PHB outlined above underlie its potential as a carrier for bioactive agents, with prospective applications in the fields of medicine and cosmetics. Electrospinning of PHB from its solutions can be readily achieved, resulting in fibrous materials with good tensile strength and Young’s modulus [18,19]. Poly(ethylene oxide) (PEO), an amphiphilic polymer, is a suitable partner for the preparation of aliphatic polyester-based fibrous materials due to its biocompatibility and its ability to impart hydrophilic properties to the resulting mats [20,21]. Furthermore, the incorporation of PEO into fibrous materials is crucial for enhancing the solubility and release of bioactive compounds with low water-solubility, such as most naturally occurring polyphenolic compounds.

The incorporation of rutin (RUT) into fibrous materials by electrospinning is of considerable interest owing to the antimicrobial, antioxidant, and anticancer properties of this plant-derived flavonoid [22]. RUT-loaded fibers have been successfully prepared using various polymer blends, including pullulan/poly(vinyl alcohol) [23], chitosan oligosaccharide/polycaprolactone [24], cellulose acetate/poly(ethylene oxide) [25], cellulose acetate/poly(ethylene glycol) [26], poly(vinyl alcohol)/Eudragit L100 [27], and hydroxyethyl cellulose/poly(vinyl alcohol) [28]. These materials have shown potential applications in the biomedical field [24,25,26,27,28], as well as in the food industry [28]. There are data on the incorporation of the flavonoid catechin or quercetin in poly(lactic acid)/PHB or PHB fibrous materials, respectively, via electrospinning with potential application as agricultural mulch films, films for food packaging or in biomedicine [29,30]. Up to our knowledge, there are no data on the fabrication of RUT-loaded PHB/PEO fibrous materials by electrospinning. Another highly promising plant-derived polymer for design of novel composite fibrous materials with potential biomedical applications is the protein zein, owing to its biocompatibility, biodegradability, and cost-effectiveness [31,32]. Due to its amphiphilic nature, zein has been demonstrated to serve as a cargo polymer for the encapsulation of poorly water-soluble bioactive compounds via electrospraying [14,33,34,35]. Up to now, there are no data on the incorporation of *Melissa officinalis* (MO) extract into zein particles by electrospraying. MO extracts possess a set of valuable biological activities, including antimicrobial, antiviral, antioxidant, and anticancer properties, which make them attractive candidates for biomedical applications. To date, only a limited number of studies have reported the development of electrospun fibrous materials containing MO extracts. MO-loaded fibrous materials have been prepared by blend electrospinning [36,37,38] or coaxial electrospinning [39]. Recently, some of us reported that electrospun PHB fibrous materials loaded with MO extract, as well as coated with polyelectrolyte complex from chitosan/hyaluronic acid PHB fibrous materials containing MO and/or *Hypericum perforatum* extract exhibited antioxidant, antibacterial, and anticancer activities [40]. These materials were obtained by electrospinning or electrospinning followed by dip coating and subsequent polyelectrolyte complex formation.

It was hypothesized that the simultaneous electrospinning/electrospraying technique would enable the preparation of nonwoven textiles with a complex architecture composed of PHB/PEO/RUT fibers decorated with zein/MO particles. Spatially separating RUT in the fibers and MO in the particles would facilitate the independent and mutually unaffected release of the bioactive compounds. Furthermore, such fibrous materials could be anticipated to exhibit targeted antioxidant and anticancer activities arising from the combined action of RUT and MO. The presence of PEO in the PHB-based composite materials was expected to impart hydrophilicity, which was important for the release of the poorly water-soluble RUT and, consequently, for enhancing its therapeutic potential against oxidative stress and cancer cells. In addition, zein might serve as an effective natural biopolymer carrier for the encapsulation of MO extract through electrospraying. Electrosprayed zein particles could provide a sustained release of the bioactive compounds from MO, which may improve their biological effectiveness. The considerations outlined above defined the aim of the present study, namely, to develop and evaluate a composite fibrous system with spatially separated incorporation of rutin (RUT) and *Melissa officinalis* extract (MO), prepared via simultaneous electrospinning and electrospraying, as well as to investigate the effect of this architecture on wettability, crystallinity, mechanical properties, release profiles of the active compounds, antioxidant activity, and selective anticancer activity against melanoma cells.

## 2. Materials and Methods

### 2.1. Materials

Poly(3-hydroxybutyrate) (PHB, Biomer, Schwalbach am Taunus, Germany, M_n_ 330,000 g/mol), poly(ethylene oxide) (PEO, Serva, Heidelberg, Germany, M_n_ 100,000 g/mol) and zein (Sigma, Saint Louis, MA, USA) were used. Rutin (RUT, 97+%) was purchased from Acros Organics, Geel, Belgium. 2,2-Diphenyl-1-picrylhydrazyl (DPPH) was bought from Sigma-Aldrich (Darmstadt, Germany); chloroform, dimethylformamide, absolute ethanol, NaH_2_PO_4_ and KH_2_PO_4_—from Merck (Darmstadt, Germany). All of the aforementioned chemicals were of analytical grade and were used without further purification. All solvents employed in the HPLC and HPLC-ESI/MS analyses were of HPLC and LC–MS grade.

Plant material from cultivated *Melissa officinalis* (MO) was bought from “Essential Oils and Herbs” Company Ltd. (Blatets Village, Bulgaria). The method used to obtain the MO dry extract has been described previously [36]. Briefly, the aerial parts of the MO species were subjected to extraction with 70% aqueous methanol for 24 h at 25 °C. Following extraction, the mixture was filtered, and the solvent was removed under reduced pressure. The remaining aqueous fraction was subjected to spray drying using a Büchi Mini Spray Dryer B-290 (BUCHI Labortechnik AG, Flawil, Switzerland), yielding the MO dry extract.

Human skin melanoma cell line SH-4 (CRL-7724) was purchased from the American Type Culture Collection (ATCC, Rockville, MD, USA). The non-cancerous human keratinocyte line HaCaT (CVCL_0038) was sourced by CLS Cell Lines Service (Eppelheim, Germany). Acridine orange (AO), ethidium bromide (EtBr), neutral red (NR) from Sigma (St Louis, MO, USA), and Dulbecco’s modified Eagle medium (DMEM) were supplied from Sigma-Aldrich, Schnelldorf, Germany. The cell culture reagents fetal bovine serum (FBS) (Gibso/BRL, Grand Island, NY, USA), L-glutamine, penicillin, and streptomycin solution (LONZA, Cologne, Germany) were used. AppliChem, Darmstadt, Germany, supplied the 4′,6-diamidino-2-phenylindole (DAPI). The plastic consumables were sourced from Orange Scientific, Braine-l’Alleud, Belgium.

### 2.2. HPLC-DAD-ESI/MS Analysis of MO Dry Extract

The main compounds of the dry extract of MO were identified using HPLC-DAD-ESI/MS analysis, carried out on Shimadzu LC-2040C 3D Nexera-i and Shimadzu LCMS 2020 (single quadrupole) (Shimadzu, Tokyo, Japan). The chromatographic separation and mass spectrometric parameters were applied as previously described [36]. The determination of rosmarinic acid content in the MO extract is conducted as previously reported [36].

### 2.3. Preparation of Fibrous Materials by Electrospinning or by Simultaneous Electrospinning and Electrospraying

#### 2.3.1. Preparation of PHB, PHB/PEO and RUT-Loaded PHB/PEO Mats by Electrospinning

For the preparation of the PHB mats, the PHB spinning solution (14% *w*/*v*) was prepared by dissolving PHB in a CHCl_3_/DMF (4/1, *v*/*v*) solvent mixture at 60 °C under reflux conditions with continuous stirring for 4 h. PHB/PEO mats with a PHB:/PEO weight ratio of 70/30 were fabricated by electrospinning from their blend solutions in CHCl_3_/DMF (4/1, *v*/*v*) (total polymer concentration of 14% *w*/*v*). To prepare RUT-loaded PHB/PEO mats (hereafter denoted as PHB/PEO/RUT), a spinning solution of PHB, PEO, and RUT was obtained by dissolving 3.92 g of PHB in 26 mL of chloroform at 60 °C, followed by the addition of a chloroform solution of PEO (1.68 g; 6 mL) and a DMF solution of RUT (0.392 g; 8 mL). The mixture was stirred for 4 h. The RUT content was 6.54 wt% relative to the total weight of the solids. For the electrospinning experiments, the solution was delivered at a feed rate of 3 mL/h. The resulting fibers were deposited onto a rotating drum collector operating at 1400 rpm, with a tip-to-collector distance of 25 cm. A voltage of 25 kV was applied using a scientific high-voltage generator (Model HVG-CONT-LCD, Linari Engineering, Pisa, Italy). All experiments were carried out at room temperature (25 °C) and a relative humidity of 50%.

#### 2.3.2. Preparation of Zein/MO-*on*-PHB/PEO/RUT and Zein/MO-*on*-PHB/PEO Mats by Simultaneous Electrospinning and Electrospraying

The electrospun PHB/PEO mats decorated with zein particles containing MO are assigned as zein/MO-*on*-PHB/PEO. The PHB/PEO/RUT fibers bearing MO-loaded zein particles on their surface are designated as zein/MO-*on*-PHB/PEO/RUT. Zein/MO-*on*-PHB/PEO and zein/MO-*on*-PHB/PEO/RUT mats were prepared by simultaneous electrospinning of PHB/PEO or PHB/PEO/RUT solution, and electrospraying of a zein/MO solution. PHB/PEO or PHB/PEO/RUT spinning solutions were prepared following the procedure described in Section 2.3.1. For electrospraying, a solution of zein and MO was obtained in 70 vol.% ethanol, with zein concentration of 5 wt.% and MO concentration of 10 wt.% relative to the zein weight. Electrospinning in conjunction with electrospraying were carried out for a deposition time of 10 h. Two separate syringes loaded with prepared PHB/PEO or PHB/PEO/RUT and zein/MO solutions were used. The syringes were mounted on two infusion pumps (NE-300 Just Infusion™ Syringe Pump, New Era Pump Systems Inc., Farmingdale, NY, USA). The pumps were positioned on the opposite sides of the collector at an angle of 180°. Additionally, electrospinning was performed under the same parameters as those described in Section 2.3.1., while electrospraying was carried out at 27 kV applied voltage from a custom-made high voltage DC power supply, a tip-to-collector distance of 13 cm with a zein/MO solution flow rate of 1 mL/h. The experiments were conducted at collector rotation speed of 1400 rpm, a temperature of 25 °C and ca. 50% relative humidity. The mats were kept under reduced pressure at 25 °C to eliminate residual solvent.

### 2.4. Characterization of the Fibrous Materials

The dynamic viscosity of PHB, PHB/PEO, PHB/PEO/RUT solutions was measured at 25 ± 0.1 °C using a Brookfield DV-II+ programmable cone/plate viscometer (Brookfield, Middleboro, MA, USA) at 6 rpm (for PHB spinning solution) or 12 rpm (for PHB/PEO and PHB/PEO/RUT spinning solutions). The corresponding shear rate was calculated as SRC × RPM, where SRC = 2 s^−1^/rpm (for spindle CPE 52), giving a shear rate of 12 s^−1^ (for PHB spinning solution) or 24 s^−1^ (for PHB/PEO and PHB/PEO/RUT spinning solutions). The electrical resistance of the solutions was measured using an electrolytic cell equipped with rectangular sheet platinum electrodes, as described in [41].

The morphology of the fibers and particles was examined using a scanning electron microscopy (SEM) with the aid of a Jeol JSM-5510 (JEOL Ltd., Tokyo, Japan) SEM microscope. Prior to imaging, the mats were coated with gold. A minimum of 30 fibers from three separate SEM images (totaling 90 fibers per mat) were analyzed using ImageJ software (v1.53e, Wayne Rasband, National Institutes of Health, Bethesda, MD, USA) to assess the morphology of the fibers and particles.

The surface wettability of the fibrous materials was assessed using an Easy Drop DSA20E Krüss drop shape analysis system (KRÜSS GmbH, Hamburg, Germany). A 10 μL sessile droplet of distilled water, delivered via a computer-controlled dosing unit, was deposited onto the mat surface. The water contact angle was calculated by computer-based analysis of the captured droplet images. All results are reported as the mean of 20 measurements for each sample.

Attenuated total reflection Fourier transform infrared (ATR-FTIR) spectra were collected using a Nicolet™ iS™50 spectrometer (Thermo Fisher Scientific Inc., Waltham, MA, USA) fitted with an IR diamond iS50 ATR accessory (diamond crystal, with an IR penetration depth of approximately 2 μm). The spectra were obtained at a resolution of 2 cm^−1^ over the range of 4000–400 cm^−1^ using OMNIC software (Version 9.13.0.1224), with corrections applied for H_2_O and CO_2_.

Thermogravimetric analysis (TGA) was performed using a PerkinElmer TGA 4000 (PerkinElmer, Waltham, MA, USA) at a heating rate of 10 °C/min under an argon atmosphere. Instrument control, data acquisition, and processing were carried out using Pyris software (v. 11.0.0.0449).

X-ray diffraction (XRD) analysis was carried out to evaluate the presence or absence of a crystalline phase in the prepared fibrous materials. XRD patterns were collected using a computer-controlled Bruker D8 Advance powder diffractometer (Bruker, Billerica, MA, USA) equipped with a filtered CuKα radiation source and a luminescent detector. Measurements were performed over a 2*θ* range of 5° to 50°, with a step size of 0.02° and a counting time of 1 s per step.

Differential scanning calorimetry (DSC) was carried out using a Discovery DSC 250 (TA Instruments, New Castle, DE, USA) to evaluate the thermal properties of the fibrous materials. Samples were heated from 20 to 800 °C at a rate of 10 °C/min under a nitrogen atmosphere. The first heating run was used to determine the melting temperature (T_m_) and the enthalpy of fusion (ΔH_m_). The degree of crystallinity of PHB (χ_c_^PHB^, %) and PEO (χ_c_^PEO^, %) in the mats was determined according to Equations (1) and (2):

(1) $$\chi_{c}^{\mathrm{PHB}},\%=\left[\frac{\left(\Delta H_{m}^{\mathrm{PHB}}\right)}{\left(\Delta H_{m}^{\mathrm{PHB},0}\times {\;W}^{\mathrm{PHB}}\right)}\right]\times 100,$$

(2) $$\chi_{c}^{\mathrm{PEO}},\%=\left[\frac{{\Delta H}_{m}^{\mathrm{PEO}}}{\left({\Delta H}_{m}^{\mathrm{PEO},0}\times {\;W}^{\mathrm{PEO}}\right)}\right]\times 100,$$

where W^PHB^ (W^PEO^) is the mass fraction of PHB (PEO) in the mats; ΔH_m_^PHB^ (ΔH_m_^PEO^) is the PHB (PEO) enthalpy of fusion in the mats; ΔH_m_^0^ is the enthalpy of fusion, when the respective polymer is in 100% crystalline state; ΔH_m_^PHB,0^ = 146.0 J/g [42]; ΔH_m_^PEO,0^ = 213.7 J/g [43].

Tensile properties of the fibrous materials, namely PHB, PHB/PEO, PHB/PEO/RUT, zein/MO-*on*-PHB/PEO, and zein/MO-*on*-PHB/PEO/RUT, were evaluated using an INSTRON 3344 system (Instron, Norwood, MA, USA) for mechanical testing equipped with a 50 N loading cell. The tests were carried out at room temperature with a stretching rate of 20 mm/min. Specimens of 20 mm × 60 mm (width × length) were prepared with their longitudinal axis parallel to the collector rotation direction. The thickness of each sample was measured using a Digital Thickness Gauge FD 50 (Käfer GmbH, Bremen, Germany). During testing, the initial gauge length was fixed at 40 mm. For each material, ten specimens were analyzed, and the average values of Young’s modulus, tensile strength, and elongation at break were calculated.

### 2.5. In Vitro RUT and MO Release

The actual RUT loading amount was evaluated by UV–vis spectrophotometry. Purposely, samples of PHB/PEO/RUT or zein/MO-*on*-PHB/PEO/RUT mats were immersed in ethanol/water = 70/30 (*v*/*v*). The actual amount of loaded RUT was estimated by Beckman Coulter DU800 UV–vis spectrophotometer at a wavelength of 359 nm. The actual loading amount of MO in the mat was estimated taking into account (*i*) the actual loading amount of rosmarinic acid from MO extract in the mat; and (*ii*) the content of rosmarinic acid in MO extract. The actual loading amount of rosmarinic acid from MO extract in the fibrous materials was studied via HPLC-DAD-ESI/MS analysis. Samples of zein/MO-*on*-PHB/PEO or zein/MO-*on*-PHB/PEO/RUT mats were immersed in ethanol/water = 70/30 (*v*/*v*). The chromatographic separation and mass spectrometry settings were performed according to the procedure reported in [36]. The experiments were performed in triplicate.

The drug loading capacity (%) and encapsulation efficiency (%) were calculated using Equations (3) and (4):

Drug loading capacity (%) = Weight of actual drug loading/Weight of the mat × 100,(3)

Encapsulation drug efficiency (%) = Weight of actual drug loading/Weight of theoretical drug loading × 100,(4)

The in vitro release profile of RUT, as well as the release profile of rosmarinic acid, one of the major bioactive compounds found in MO extract, was studied in PBS solution containing Tween 80 (pH 7.4; PBS/Tween 80 = 99.2/0.8 *v*/*v*; buffer ionic strength of 0.1) at 37 °C. The fibrous mats (24 mg) were immersed in 40 mL of the prepared PBS/Tween 80. They were maintained at 37 °C in a thermostatically controlled shaking water bath (JULABO SW23, JULABO GmbH, Allentown, PA, USA) with continuous agitation at 100 rpm. At specific intervals of time, 2 mL aliquots were collected. The removed volume was then replaced with fresh buffer solution.

The amount of the released RUT was determined by DU 800 UV–vis spectrophotometer produced by Beckman Coulter, Brea, CA, USA, at a wavelength of 361 nm. The amount of the released rosmarinic acid from MO extract was estimated using HPLC-DAD-ESI/MS analysis. The chromatographic separation and mass spectrometry settings were carried out according to the procedure reported in [36]. The cumulative release percentage of RUT and rosmarinic acid was determined as a function of time. The results shown represent the mean values obtained from three independent measurements.

### 2.6. Antioxidant Capacity

The DPPH^•^ assay was utilized to evaluate the radical-scavenging activity of the mats and solutions. A 0.5 mL solution of RUT (0.075 mg) or MO (0.025 mg) in an ethanol was mixed with 3.0 mL of a 0.1 × 10^−3^ M DPPH^•^ solution in ethanol. Fibrous samples from PHB/PEO/RUT, PHB/PEO, PHB, zein/MO-*on*-PHB/PEO, or zein/MO-*on*-PHB/PEO/RUT containing 0.075 mg RUT and/or 0.025 mg MO were placed in 0.5 mL of ethanol, followed by the addition of 3.0 mL of a 0.1 × 10^−3^ M DPPH^•^ solution in ethanol. All solutions were kept in the dark at 24 °C for 30 min. The absorbance of the DPPH^•^ solution at 517 nm was measured after the addition of the solutions or mats, using a DU 800 UV–Vis spectrophotometer (Beckman Coulter, Brea, CA, USA). The following equation was utilized to calculate the antioxidant activity (AA%):

Inhibition, AA, % = [(A_DPPH_^•^ − A_sample_)/A_DPPH_^•^] × 100, (5)

where A_DPPH_^•^ denotes the absorbance of the DPPH^•^ solution, while A_sample_ refers to the absorbance of the DPPH^•^ solution after adding the tested sample. Each experiment was carried out three times.

### 2.7. Neutral Red Uptake (NRU) Assay

Antiproliferative activity and potential cytotoxic effects of the tested fibrous materials on SH-4 melanoma cells and HaCaT non-cancerous keratinocytes were evaluated by the standard NRU assay. It is a spectrophotometric method based on the ability of viable cells to incorporate and bind the cationic dye neutral red in lysosomes. The amount of dye taken up is proportional to the number of viable cells. Briefly, cells were plated in 96-well plates at a density of 1 × 10^4^ cells per well (100 µL) and incubated for 24 h before treatment with RUT, MO, and different fibrous materials. RUT and MO were applied at concentrations ranging from 3.125 to 400 µg/mL and 15.625 to 2000 µg/mL, respectively, for 24 and 48 h. Each concentration was tested in six replicates. Different fibrous materials (PHB, PHB/PEO, PHB/PEO/RUT, zein/MO-*on*-PHB/PEO, and zein/MO-*on*-PHB/PEO/RUT) were also tested in six repetitions per sample for 24 and 48 h. All fibrous materials loaded with RUT and/or MO contained RUT and MO at concentrations of 300 μg/mL and 250 μg/mL, respectively. The respective untreated cells cultured in medium alone served as negative controls. At the completion of the treatment, the medium was discarded and NRU solution was added to all wells, and the plates were additionally incubated for 3 h at 37 °C. Finally, the dye was eluted with ethanol/acetic acid solution. The absorbance was measured at 540 nm wavelength on a TECAN microplate reader (TECAN, Grödig, Austria), and cell viability was expressed as a percentage relative to untreated controls. The concentrations required for 50% inhibition (IC_50_) of cell growth were calculated using nonlinear regression analysis (GraphPad Prism 4 software).

### 2.8. Assessment of Apoptosis Using Fluorescent Staining Methods

#### 2.8.1. Dual Fluorescent Staining with AO/EtBr

Morphological analysis of cell apoptosis was performed using acridine orange/ethidium bromide (AO/EtBr) dual staining. In brief, SH-4 cancer cells and HaCaT non-cancerous cells (1 × 10^5^ cells/well) were cultured on 13 mm diameter cover glasses in 24-well plates overnight. After reaching confluence, the cells were incubated with the tested fibrous materials (PHB, PHB/PEO, PHB/PEO/RUT, zein/MO-*on*-PHB/PEO, and zein/MO-*on*-PHB/PEO/RUT) and with RUT and MO solutions for 24 h. Cells cultured in medium alone served as controls. Following incubation, coverslips were removed, rinsed with PBS and then stained with fluorescent dyes AO and EtBr (5 µg/mL each in PBS) for 5 min. The stained cells were immediately observed using a Leica DM 5000B fluorescence microscope (Leica Microsystems, Wetzlar, Germany) at 40× magnification.

#### 2.8.2. DAPI Fluorescent Staining

The DNA-binding dye 4′,6-diamidino-2-phenylindole (DAPI) was used to identify apoptotic cells through the detection of characteristic nuclear morphological changes associated with apoptosis. DAPI effectively visualizes nuclear DNA in both live and fixed cells because it can penetrate intact cytoplasmic membranes. The cells were cultured on glass coverslips and treated with the tested samples, as described in the previous section. After the incubation period, the cells were fixed with 3% paraformaldehyde for 10 min, rinsed with PBS, and incubated with a 1 µg/mL DAPI solution for 15 min at room temperature in the dark. The samples were then covered with Mowiol^®^ and mounted on slides. Apoptotic nuclei were observed under a Leica DM 5000B fluorescence microscope (Wetzlar, Germany) using a DAPI filter.

### 2.9. Statistical Analysis

All experiments were conducted in triplicate, and the data are expressed as mean values with their corresponding standard deviations (±SD). Statistical significance was assessed using one-way analysis of variance (ANOVA), followed by Bonferroni’s post hoc test. All statistical analyses were performed with GraphPad PRISM software (Version 5; GraphPad Software Inc., San Diego, CA, USA). A *p*-value of less than 0.05 (*p* < 0.05), 0.01 (*p* < 0.01), and 0.001 (*p* < 0.001) was regarded as statistically significant.

## 3. Results and Discussion

### 3.1. Main Phenolic Compounds and Total Rosmarinic Acid Content in MO Dry Extract

Previous analyses of the MO dry extract employing HPLC-DAD-ESI/MS [34] demonstrated the presence of phenolic acids, including rosmarinic, caffeic, caftaric, sagerinic, sulfated rosmarinic, and salvianolic acids, whereas luteolin 7-O-glucuronide was identified as the only flavonoid present in MO. Among these compounds, rosmarinic acid was the predominant constituent, with a content of 76.27 ± 0.1 mg/g (determined by HPLC-DAD). This compound is considered to significantly contribute to the biological activity of MO extracts [44].

### 3.2. Morphology of Fibrous Materials

The combination of the favorable properties of the aliphatic polyester PHB, the water-soluble polymer PEO, and the plant protein zein, together with the flavonoid rutin (RUT) and MO extract—both known for their antioxidant, antibacterial, and anticancer activities—offers a promising approach for the design of novel materials with potential applications in various biomedical fields.

**Scheme 1:**
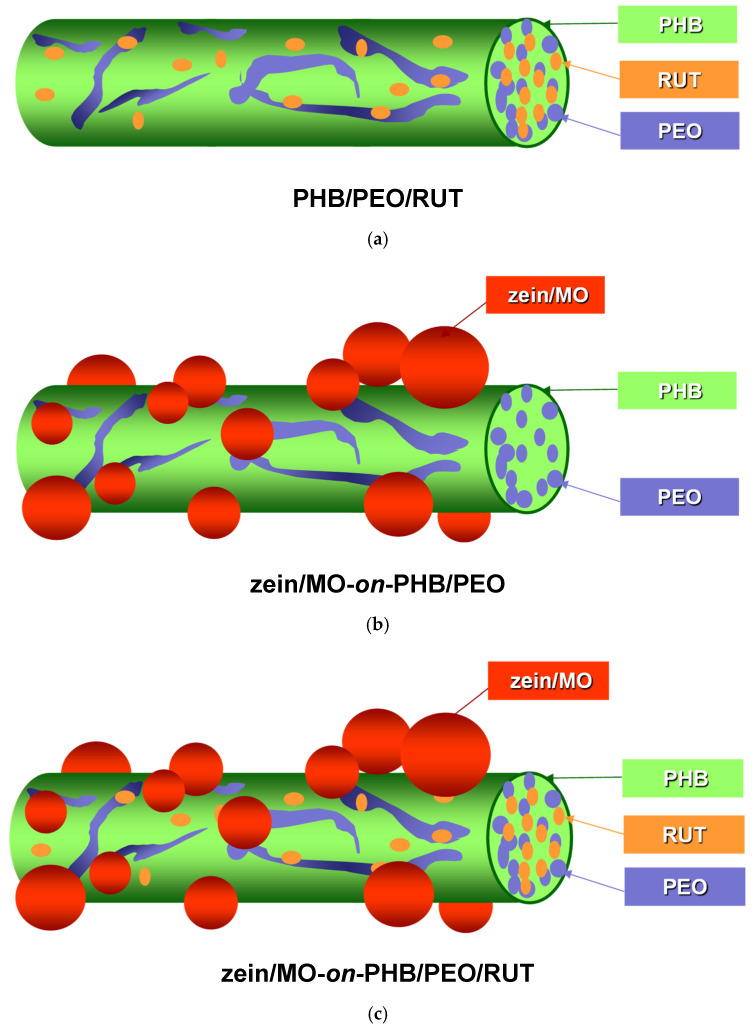
Schematic representation of fibers prepared by electrospinning (**a**) or simultaneous electrospinning and electrospraying (**b**,**c**).

A schematic representation of fibers from the three types of innovative fibrous materials loaded with RUT and/or MO extract, featuring diverse architecture, obtained in the present study, is presented in Scheme 1. To obtain them in one step, two different approaches were used, namely: (i) electrospinning of mixtures of PHB, PEO, and RUT (PHB/PEO/RUT mat, RUT content 6.54 wt% relative to the total weight of the solids; Scheme 1a), and (ii) simultaneous electrospinning of PHB/PEO or their mixtures with RUT, and electrospraying of a zein/MO solution (zein/MO-*on*-PHB/PEO mat, MO content 2.06 wt% relative to the total weight of the solids; or zein/MO-*on*-PHB/PEO/RUT mat, RUT and MO content 5.83 and 1.94 wt%, respectively, relative to the total weight of the solids, Scheme 1b,c). Fibrous materials from PHB and PHB/PEO were also obtained (Supplementary Materials, Scheme S1).

**Figure 1:**
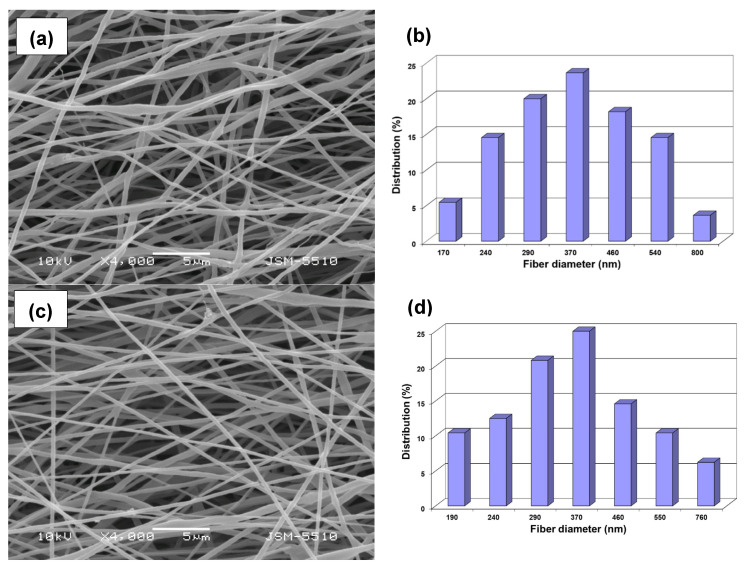

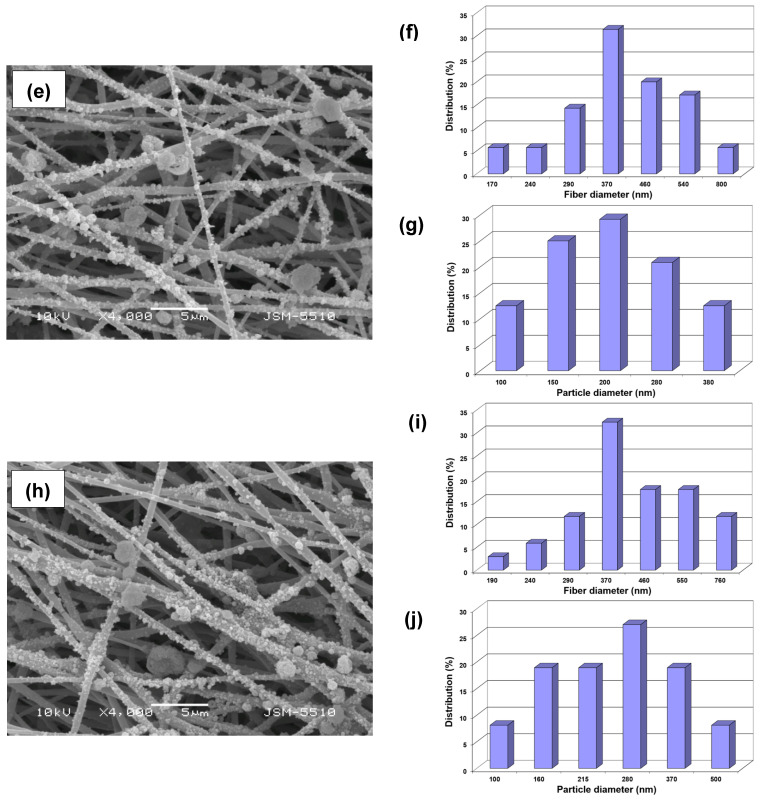
SEM micrographs (**a**,**c**,**e**,**h**), fiber diameter distributions (**b**,**d**,**f**,**i**), and particle diameter distributions (**g**,**j**) of the prepared electrospun mats: (**a**,**b**) PHB/PEO, (**c**,**d**) PHB/PEO/RUT, (**e**–**g**) zein/MO-*on*-PHB/PEO and (**h**–**j**) zein/MO-*on*-PHB/PEO/RUT. Magnification × 4000.

Scanning electron microscopy (SEM) was used to assess the morphology of the prepared fibrous materials. Figure 1 represents the morphology and diameter distributions of the fibers and/or particles in the fibrous materials. As seen, the electrospinning of a solution from PHB/PEO/RUT (total polymer concentration: 14% *w*/*v*, content of RUT: 6.54 wt% relative to the total weight of the solids) in a CHCl_3_/DMF (4/1, *v*/*v*) resulted in the preparation of fibers with a cylindrical shape and without defects (Figure 1c). The same electrospinning conditions were used to obtain cylindrical fibers from PHB and PHB/PEO without defects (Supplementary Materials, Figure S1 and Figure 1a). The PHB fibers possessed a mean fiber diameter of 495 ± 180 nm (Supplementary Materials, Table S1). An insignificant drop in the mean fiber diameter was detected upon the addition of PEO to the PHB spinning solution: from 495 ± 180 nm for PHB to 380 ± 140 nm for PHB/PEO mats. A decrease in the viscosity of the PHB solution with the addition of PEO with lower molar mass was registered (Supplementary Materials, Table S1). The addition of RUT to the spinning solution of PHB/PEO also led to a slight decline in the mean fiber diameter (370 ± 145 nm). This fact is likely attributed to a slight decrease in the viscosity of the RUT-containing solution compared to the PHB/PEO solution (Supplementary Materials, Table S1).

The deposition of zein/MO particles on the surface of PHB/PEO or PHB/PEO/RUT fibers, as well as in the voids between the fibers, was observed for fibrous materials prepared by electrospinning in conjunction with electrospraying (Figure 1e,h). A small number of zein/MO particle aggregates was detected along the length of the fibers. As seen, the particles deposited on the PHB/PEO or PHB/PEO/RUT fibers exhibited a spherical shape. The mean particle size in the case of zein/MO-*on*-PHB/PEO and zein/MO-*on*-PHB/PEO/RUT were 210 ± 100 nm and 270 ± 112 nm, respectively. In the case of mats decorated with zein/MO particles a rough surface morphology was registered. These findings are in conformity with previous reports [9,36,45]. The obtained SEM results demonstrated the successful fabrication of mats loaded with RUT and/or decorated with zein/MO particles via electrospinning or electrospinning combined with electrospraying.

### 3.3. Wettability of the Fibrous Materials

**Figure 2:**
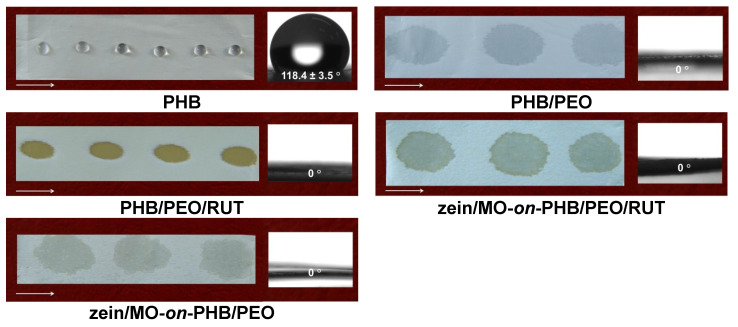
Digital images showing water droplets deposited on the mat surface. The white arrow denotes the direction of rotation of the collector.

The adhesion and proliferation of cancer cells are greatly affected by the hydrophilic/hydrophobic characteristics of the fibrous mats [46]. Accordingly, the contact angles of the fabricated materials loaded with RUT, MO, or both were measured to assess their wettability. As presented in Figure 2, the PHB fibrous materials was hydrophobic with a water contact angle (WCA) of 118.4 ± 3.5 °C. The observed hydrophobicity is mainly attributed to the non-polar chemical structure of PHB, the limited availability of surface-exposed polar groups, and the predominance of hydrophobic hydrocarbon segments at the fiber surface.

Adding 30 wt% of the water-soluble polymer PEO imparts wettability to the mats surface (Figure 2). Upon deposition of water droplets, the WCA of the PHB/PEO mats immediately decreased to 0°. The prompt decrease in WCA is primarily attributed to the fast hydration of PEO, which leads to rapid swelling and partial redistribution of PEO-rich domains on the fiber surface immediately after water deposition. This facilitates immediate spreading and absorption of the water droplet. The porous structure of the electrospun mats also plays role in the detected rapid absorption of the water droplets. The obtained result is consistent with previously reported data on electrospun materials containing PEG or PEO [47,48]. Similarly, all other PEO-containing fibrous materials loaded with RUT and/or MO showed hydrophilic behavior and a WCA value of 0° (Figure 2). The multiple hydroxyl groups in RUT and MO extract constituents can also facilitate the wettability of the fibrous materials. Wettability is considered an important characteristic for enabling fast therapeutic effectiveness of the incorporated bioactive compounds, especially in view of the potential biomedical use of the developed materials.

### 3.4. ATR-FTIR Spectra of the Fibrous Materials

**Figure 3:**
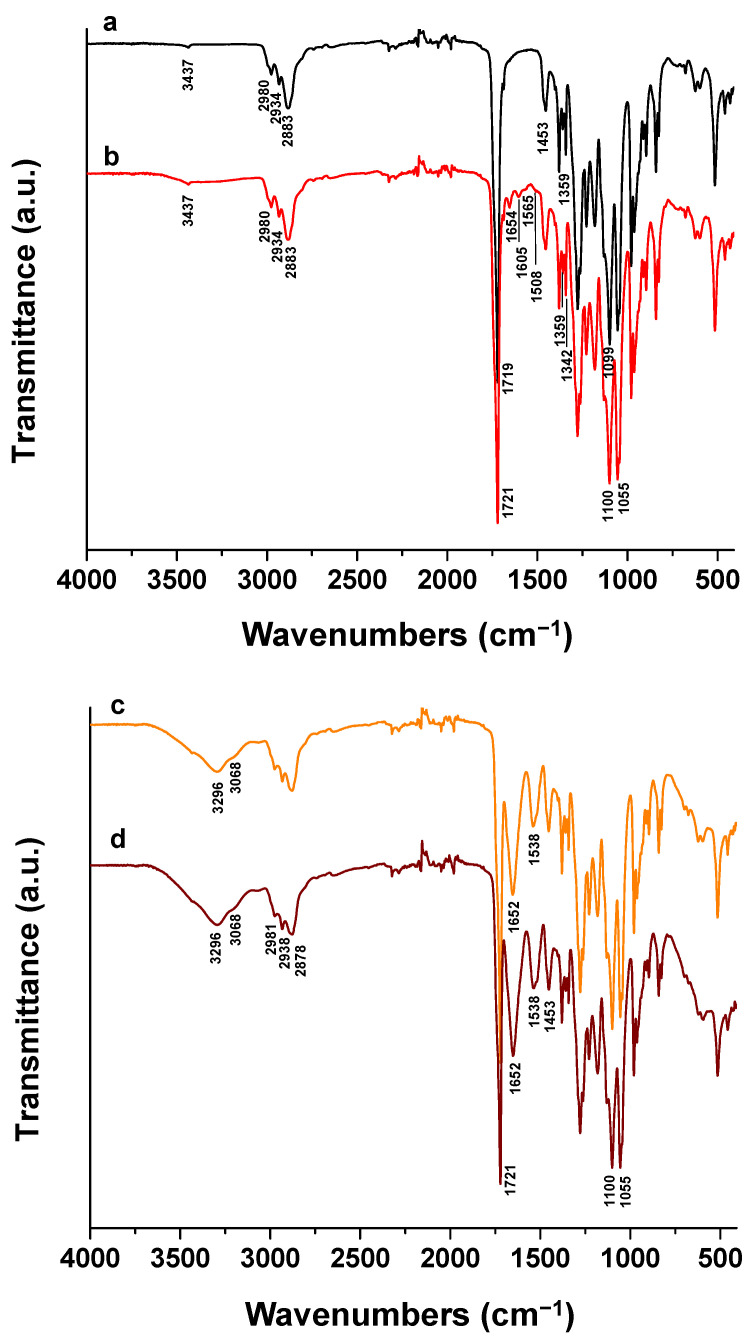
ATR–FTIR spectra of mats: (a) PHB/PEO, (b) PHB/PEO/RUT, (c) zein/MO-*on*-PHB/PEO, and (d) zein/MO-*on*-PHB/PEO/RUT.

ATR-FTIR spectroscopy was utilized to confirm the chemical structure of PHB/PEO mats loaded with RUT and/or MO. The spectrum of PHB/PEO mats displayed characteristic bands at 1099 cm^−1^ related to C-O-C stretching from PEO and at 1342 cm^−1^ assigned to C-H bending vibrations of PEO with the exception of the bands of PHB (3437 cm^−1^ ascribed to O-H stretching vibrations; 2980, 2934, and 2883 cm^−1^, assigned to C-H aliphatic stretching; 1719 cm^−1^ characteristic for C=O stretching; 1453 cm^−1^, ascribed to C-H bending vibrations of the polymer chain of PHB; 1055 cm^−1^ for C-O-C stretching) (Figure 3(a)). Additionally, in the spectrum of PHB/PEO mat compared to those of PHB mat (Supplementary Materials, Figure S2a), a rise in the intensity of the bands at 2980, 2934, and 2883 cm^−1^ which corresponds to aliphatic C-H stretching vibrations, was registered. As seen in Figure 3(b), in the case of the spectrum of RUT-loaded PHB/PEO mats, new bands at 1605, 1565 and 1508 cm^−1^ ascribed to the stretching vibrations of the benzene ring of RUT appeared [49]. A new band at 1654 cm^−1^ characteristic for carbonyl stretching of RUT [49] was also detected. Furthermore, a broad band of minor relevance at 3437 cm^−1^ attributed to the stretching vibrations of O-H from PHB and RUT was registered in the spectrum of the RUT-loaded PHB/PEO mats. In the PHB/PEO/RUT mat spectrum (Figure 3(b)), characteristic bands were shifted compared to those of PHB/PEO mat (Figure 3(a)): the band assigned to C=O stretching from PHB at 1719 cm^−1^ is shifted by 2 cm^−1^ toward higher wavenumbers, the band ascribed to C-O-C stretching of PEG at 1099 cm^−1^ is shifted by 1 cm^−1^ toward higher wavenumbers. Accordingly, it can be inferred that interactions most probably based on hydrogen bonds are established between the PHB or PEO and RUT.

As seen in Figure 3(c,d), in the spectra of PHB/PEO or PHB/PEO/RUT mats, decorated with zein/MO particles additional bands were observed alongside those of PHB and PEO. These new intensive peaks appeared at 1652 cm^−1^, corresponding to stretching vibrations of the C=O and C-N groups in amide I from zein; at 1538 cm^−1^, associated with both C-N stretching vibrations and N-H bending vibrations in amide II from zein; and at 3296 cm^−1^ and 3068 cm^−1^, which can be attributed to O-H and N-H stretching vibrations from zein [50]. An increase in the intensity of the band at 1453 cm^−1^ was observed, most likely due to the contribution of vibration of the -CH group in amide III from zein [50]. In these spectra, the bands observed at 1652 cm^−1^ and 1538 cm^−1^ were shifted to higher wavenumbers by 8 cm^−1^ and 22 cm^−1^, respectively, relative to the corresponding bands of zein powder located at 1644 cm^−1^ and 1516 cm^−1^ (Supplementary Materials, Figure S2c). In addition, a shift in the band at 3296 cm^−1^ toward a higher wavenumber by 4 cm^−1^ was also registered compared to its position in the zein spectrum at 3292 cm^−1^. The presence of these bands shifts suggests the formation of hydrogen bonds between zein and the MO extract. The performed ATR-FTIR analyses also confirmed the successful incorporation of RUT into PHB/PEO fibers and the successful deposition of zein/MO particles onto the fiber surface.

### 3.5. TGA Analyses of the Fibrous Materials

Further evidence for the successful fabrication of zein/MO-*on*-PHB/PEO and zein/MO-*on*-PHB/PEO/RUT composite fibrous materials was found in their TGA thermograms (Supplementary Materials, Figure S3). Figure S3 (Supplementary Materials) also presents the thermograms of a PHB mat, PEO powder, zein powder, as well as PHB/PEO and PHB/PEO/RUT mats. The PHB mat underwent thermal degradation in one step, with a maximum degradation temperature (T_d_^max^) of 290 °C. Both the thermal degradation profile and the T_d_^max^ value are consistent with data reported by other authors [51]. PEO likewise degraded in one step, exhibiting a T_d_^max^ of 430 °C, in agreement with previously reported results [52]. Thermal degradation of the PHB mat and PEO powder proceeded without any detectable residue after heating to 800 °C. For the PHB/PEO mat prepared at a weight ratio of the polymer components 70/30, thermal degradation occurred in two distinct stages. The first stage, characterized by a T_d_^max^ of 290 °C, was attributed to the degradation of PHB present in the mat, whereas the second stage, with a T_d_^max^ of 430 °C, corresponded to the degradation of PEO in the mat. Based on the weight losses detected during the first and second degradation stages, it was calculated that the weight content of PHB in the fibrous material was 70%, while that of PEO was 30%, which correspond to the initial amounts of the polymer components in the spinning solution subjected to electrospinning. As shown in Figure S4 (Supplementary Materials), RUT undergoes thermal degradation through several stages and there is a residue of approximately 30% after heating to 800 °C. The incorporation of 6.54 wt.% RUT, relative to the total weight of the solids, in PHB/PEO/RUT electrospun materials did not affect the T_d_^max^ values during the thermal degradation of the PHB and PEO components, which remained at 290 °C and 430 °C, respectively (Supplementary Materials, Figure S3). However, a residue of ca. 1.7% was detected after heating to 800 °C, which was attributed to the presence of RUT in the mat. Similar to RUT powder, MO powder also degraded in multiple stages and left a residue of ca. 40% after heating to 800 °C (Supplementary Materials, Figure S4). As reported previously [53], zein undergoes thermal degradation in two stages: the first stage, up to approximately 150 °C, is associated with moisture evaporation, whereas the second stage (from 250 to 800 °C) corresponds to thermal degradation of the protein, with a T_d_^max^ of approximately 330 °C. The detected residue after heating to 800 °C was approximately 15%. The thermal degradation of the zein/MO-*on*-PHB/PEO composite materials, prepared via simultaneous electrospinning and electrospraying, was characterized by three degradation stages with T_d_^max^ values at 290 °C, 330 °C, and 430 °C, corresponding to PHB, zein, and PEO, respectively. The presence of these three degradation stages indicates that the zein/MO-*on*-PHB/PEO composite mats are composed of all three polymers. A residue of ca. 2% after heating to 800 °C is attributed to the presence of zein and MO in the mat composition. The zein/MO-*on*-PHB/PEO/RUT fibrous materials were likewise shown to contain PHB, zein, and PEO, as evidenced by the three recorded T_d_^max^ values of 290 °C, 330 °C, and 430 °C, corresponding to the thermal degradation of the three polymers. For these mats the residue (approximately 7%) was higher compared to the zein/MO-*on*-PHB/PEO fibrous materials, which was attributed to the presence of not only zein and MO but also RUT in the bulk of the zein/MO-*on*-PHB/PEO/RUT fibers. Overall, the TGA results provided clear evidence for the successful fabrication of zein/MO-*on*-PHB/PEO and zein/MO-*on*-PHB/PEO/RUT fibrous materials via simultaneous electrospinning of PHB/PEO or PHB/PEO/RUT solutions and electrospraying of a zein/MO solution.

### 3.6. XRD Patterns of the Fibrous Materials

**Figure 4:**
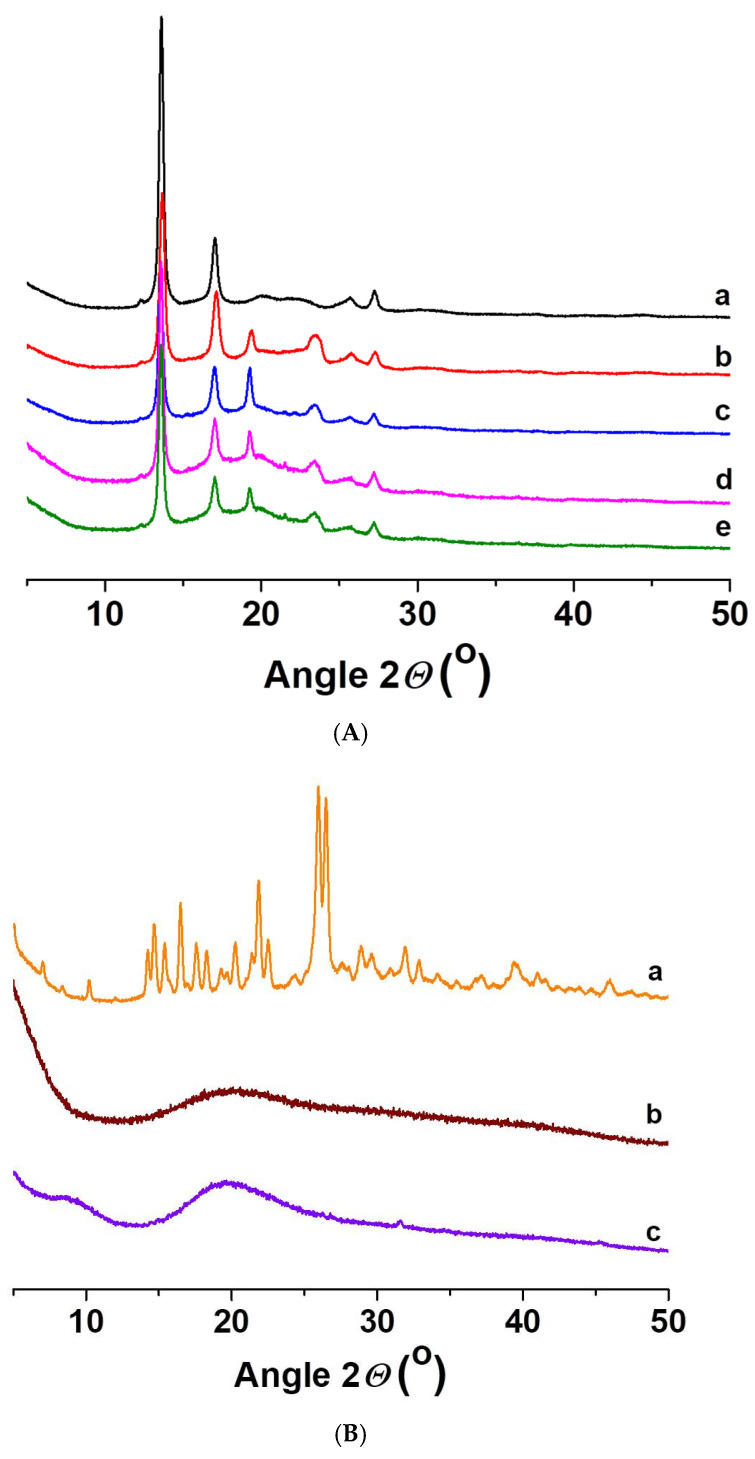
XRD patterns of mats from: (**A**) (a) PHB, (b) PHB/PEO, (c) PHB/PEO/RUT, (d) zein/MO-*on*-PHB/PEO, (e) zein/MO-*on*-PHB/PEO/RUT; and (**B**) (a) RUT powder, (b) MO powder, and (c) zein powder.

Since crystallinity is known to affect drug release behavior, XRD analysis was performed to investigate the crystallinity of the fibrous materials. The XRD patterns of the fabricated fibrous materials loaded with RUT, MO or both are displayed in Figure 4A.

As seen, the XRD patterns of PHB/PEO/RUT, zein/MO-*on*-PHB/PEO, and zein/MO-*on*-PHB/PEO/RUT mats exhibited diffraction peaks corresponding to the crystalline phase of PHB at 2*θ* values of 13.6°, 17.0°, 20.1°, 22.2°, 25.7°, and 27.2°. In addition, weak diffraction peaks associated with PEO were observed at 2*θ* values of 19.2° and 23.4°. These findings indicate that the deposition of zein/MO particles on the fiber surface did not interfere with the crystallization behavior of the PHB/PEO fibrous materials. The absence of diffraction peaks of the MO extract and zein in the XRD patterns suggests that both compounds are amorphous (Figure 4B(b,c)). Contrary, RUT powder displayed sharp diffraction peaks (2*θ* = 10.2°, 14.7°, 15.4°, 16.5°, 18.3°, 22.0°, 26.0° and 26.5°). These observations are in agreement with data reported by other authors [54]. No diffraction peaks other than those attributed to the crystalline structure of PHB and PEO were detected in the XRD patterns of the prepared fibrous mats loaded with RUT and/or MO. It is likely that, upon incorporation into the fibers from PHB/PEO by electrospinning, RUT did not undergo crystallization. This is attributed to the rapid solvent evaporation during the electrospinning process as well as steric hindrance by PHB and PEO polymer chains. The low-molecular-weight compounds present in the MO extract most probably remained in an amorphous state after being incorporated into the zein particles deposited onto the fibers. XRD data confirmed the presence of crystalline phases corresponding to PHB and PEO within the fibrous materials, while zein and the bioactive components RUT and MO are in an amorphous state.

### 3.7. DSC Thermograms of the Fibrous Materials

The thermal properties of PHB, PHB/PEO, PHB/PEO/RUT, zein/MO-*on*-PHB/PEO, and zein/MO-*on*-PHB/PEO/RUT mats were studied by DSC. The corresponding thermograms are presented in Figure S5 (Supplementary Materials). For comparison, the thermogram of PEO powder is also shown. The PEO powder exhibited a melting temperature (T_m_) at 70 °C and a degree of crystallinity of 97%. The PHB mat had T_m_ at 170 °C, with a polymer crystallinity of 64%. For the PHB/PEO mat prepared at a weight ratio of polymer components 70/30, two distinct melting peaks corresponding to PEO and PHB, respectively, were registered. This finding indicates that the two polymer components are immiscible in the blend fibers. As seen from Figure S5 (Supplementary Materials), the melting peak associated with the PEO component was shifted to a lower temperature (63 °C), whereas the T_m_ of PHB remained unchanged at 170 °C. In agreement with previous reports on the thermal behavior of fibrous materials from PHB homopolymer or its copolymer with valerate blended with PEO or PEG [55,56], the lower T_m_ of the PEO component is attributed to the formation of imperfect PEO crystals in the fibers. Regarding the crystallinity of the polymer components in the blend fibers, the degree of crystallinity of PEO was determined to be 70%, while that of PHB was 67%. These results are consistent with the diffraction reflections observed for both polymer components in the XRD patterns of the PHB/PEO mats (Figure 4A). As shown in Figure S6A (Supplementary Materials) and consistent with the XRD data (Figure 4B), RUT powder exhibits a crystalline structure, with a T_m_ of 175 °C. The incorporation of RUT into PHB/PEO/RUT fibers by electrospinning resulted in preparation of a nonwoven textile whose DSC thermogram did not display a melting peak corresponding to RUT. This finding indicates that RUT is present in an amorphous state in the fibrous material. The incorporation of RUT into PHB/PEO/RUT fibers led to a further decrease in the T_m_ of the PEO (to 59 °C), while the degree of crystallinity of PEO increased to 80% compared with that of the PHB/PEO mat. It was also found that the presence of RUT did not affect the crystallinity degree of the PHB component, which remained 67%, similar to that observed for the PHB/PEO mat. These results are consistent with data reported by other authors [56]. The DSC thermogram of zein is presented in Figure S6B (Supplementary Materials). Zein is an amorphous polymer characterized only by a glass transition temperature (T_g_) [57]. The T_g_ of the zein used in the present study was determined to be 130 °C. MO is also known to be an amorphous powder [50], which is in agreement with the amorphous halo observed for MO in the performed XRD analysis. The presence of electrosprayed zein particles in zein/MO-*on*-PHB/PEO mats could not be detected by DSC because the temperature region corresponding to the T_g_ of zein overlaps with the onset of the PHB melting peak. Regarding the effect of zein/MO particles on the thermal behavior of the semicrystalline polymers PHB and PEO in zein/MO-*on*-PHB/PEO and zein/MO-*on*-PHB/PEO/RUT mats, the particles were found to have no effect on either the melting temperatures or the crystallinity degrees of the polymers. Furthermore, RUT was in an amorphous state in the zein/MO-*on*-PHB/PEO/RUT mats, as evidenced by the absence of a melting peak corresponding to RUT in the DSC thermogram. The DSC results demonstrated the presence of crystalline phases of the semicrystalline polymers PHB and PEO in PHB/PEO, PHB/PEO/RUT, zein/MO-*on*-PHB/PEO, and zein/MO-*on*-PHB/PEO/RUT mats. In contrast, both RUT and MO were in an amorphous state in the fibrous materials. The amorphous state is of particular importance for the release of these biologically active compounds under physiological conditions.

### 3.8. Tensile Performance of the Fibrous Materials

**Figure 5:**
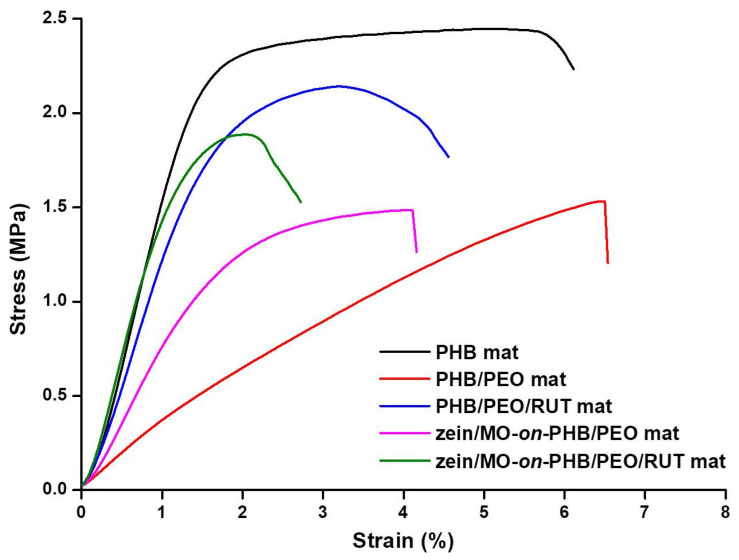
Stress–strain curves of fibrous materials from: PHB, PHB/PEO, PHB/PEO/RUT, zein/MO-*on*-PHB/PEO and zein/MO-*on*-PHB/PEO/RUT.

In order to evaluate the effect of the composition of the zein/MO-*on*-PHB/PEO and zein/MO-*on*-PHB/PEO/RUT composite fibrous materials on their mechanical performance, tensile tests were conducted. The corresponding stress–strain curves are presented in Figure 5. For comparison, the curves of PHB, PHB/PEO, and PHB/PEO/RUT mats are also shown. It was found that the PHB mat exhibited the highest tensile strength and Young’s modulus but showed brittle behavior, with an elongation at break of ca. 6% (Supplementary Materials, Table S2). Incorporation of PEO into PHB/PEO mats at a PHB/PEO weight ratio of 70/30 resulted in a substantial decrease in both tensile strength and Young’s modulus, while the elongation at break remained comparable to that of the neat PHB mat (Supplementary Materials, Table S2). As seen from Figure 5 and Table S2 (Supplementary Materials), the PHB/PEO/RUT fibrous materials exhibited higher tensile strength and Young’s modulus than the PHB/PEO mats. This improvement might be attributed to the higher crystallinity degree of PEO in the PHB/PEO/RUT mats (80%) compared with that in the PHB/PEO mats (70%), as determined by DSC analysis.

The lower elongation at break of the PHB/PEO/RUT fibrous samples compared to the PHB/PEO mats might also be attributed to the higher crystallinity degree of PEO. The zein/MO-*on*-PHB/PEO fibrous materials, fabricated by simultaneous electrospinning and electrospraying, exhibited a tensile strength comparable to that of the PHB/PEO mats; however, they showed a higher Young’s modulus and a lower elongation at break (Supplementary Materials, Table S2). These differences in the mechanical behavior of the zein/MO-*on*-PHB/PEO mats are attributed to the presence of zein/MO particles on the fiber surfaces and in the voids between the fibers. A similar trend was observed when comparing the zein/MO-*on*-PHB/PEO/RUT mat with the PHB/PEO/RUT mat (Supplementary Materials, Table S2). The presence of zein/MO particles in the zein/MO-*on*-PHB/PEO/RUT mats had no significant effect on tensile strength but resulted in an increase in Young’s modulus and a decrease in elongation at break. The obtained results demonstrate that the mechanical properties of the fibrous materials are strongly dependent on both their composition and microstructure, specifically on whether the mats consist solely of fibers or represent a composite of fibers, in which particles are deposited on the fiber surfaces or located in the voids between the fibers via simultaneous electrospinning/electrospraying.

### 3.9. In Vitro RUT and MO Release Studies

It was found that RUT loading capacity was 6.54% and 5.83% for PHB/PEO/RUT mat and zein/MO-*on*-PHB/PEO/RUT mat, respectively. The loading capacity of the rosmarinic acid (the main constituent of MO extract) was estimated to be 0.16% and 0.15% for zein/MO-*on*-PHB/PEO mat and zein/MO-*on*-PHB/PEO/RUT mat, respectively. Therefore, the loading capacity of MO was calculated to be 2.06% and 1.94% for zein/MO-*on*-PHB/PEO mat and zein/MO-*on*-PHB/PEO/RUT mat, respectively. It was found that the encapsulation efficiency of RUT and/or MO was 99.8 ± 0.1% which demonstrated that the actual drug loading is very close to the theoretical one.

**Figure 6:**
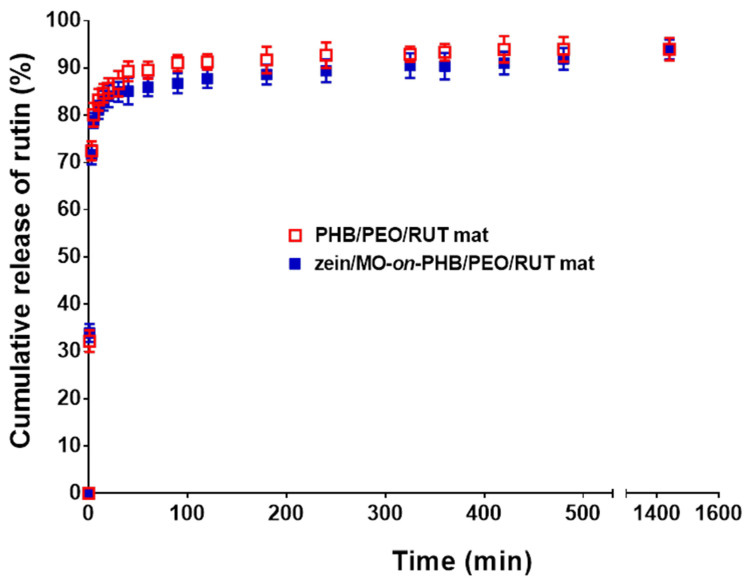
In vitro study of the release profiles of RUT from the mats: zein/MO-*on*-PHB/PEO/RUT and PHB/PEO/RUT, in PBS/Tween 80 (99.2/0.8 *v*/*v*); pH 7.4; 37 °C.

The in vitro release profiles of RUT and rosmarinic acid from MO extract from PHB/PEO mats containing RUT, MO or both compounds were studied in PBS (pH 7.4) in the presence of Tween 80 (PBS/Tween 80 volume ratio 99.2/0.8) at 37 °C, and the release time was 1440 min and 2160 min for RUT and rosmarinic acid from MO, respectively. Tween 80 is a nonionic surfactant commonly used to enhance the solubility of poorly water-soluble compounds. The release of RUT from RUT-loaded mats was monitored by UV–Vis spectrophotometry. It is known that for 1440 min PHB, PEO and zein does not undergo any hydrolytic degradation in PBS (pH 7.4) at 37 °C [58,59,60]. Thus, interference of PHB, PEO and zein degradation products on UV-vis absorption of RUT was not detected during the in vitro release studies by UV–Vis spectrophotometry. As presented in Figure 6, the in vitro release profiles of RUT from PHB/PEO/RUT and zein/MO-*on*-PHB/PEO/RUT mats were very similar. Both fibrous materials exhibited an initial burst release phase followed by a slower, gradual release stage. A plateau was detected after 1440 min. Around 85.7% and 84.0% of RUT was released from the PHB/PEO/RUT and zein/MO-*on*-PHB/PEO/RUT mats in the first 20 min, respectively. After 1440 min, the cumulative amount of released RUT increased to 94.0% and 93.9%, respectively. These release profiles indicate that the developed fibrous materials are potential delivery systems for bioactive compounds, enabling a rapid biological response. The in vitro release studies further demonstrated that the incorporation of the water-soluble polymer PEO into the fibrous matrices enhanced water penetration into the fibers and promoted the release of RUT. These findings are consistent with previous studies [61,62,63], which have also demonstrated an increased drug release rate following the incorporation of water-soluble polymers into fibrous materials based on hydrophobic biodegradable aliphatic polyesters.

The detected initial burst release of RUT is essential for achieving an effective early-stage inhibition of cancer cell proliferation. This feature is particularly important for the potential application of RUT-containing fibrous materials in local anticancer therapy, where rapid availability of the active compound may contribute to the initial suppression of cancer cells. The subsequent gradual release phase provides a sustained supply of RUT, which may contribute to the inhibition of residual cancer cell proliferation.

**Figure 7:**
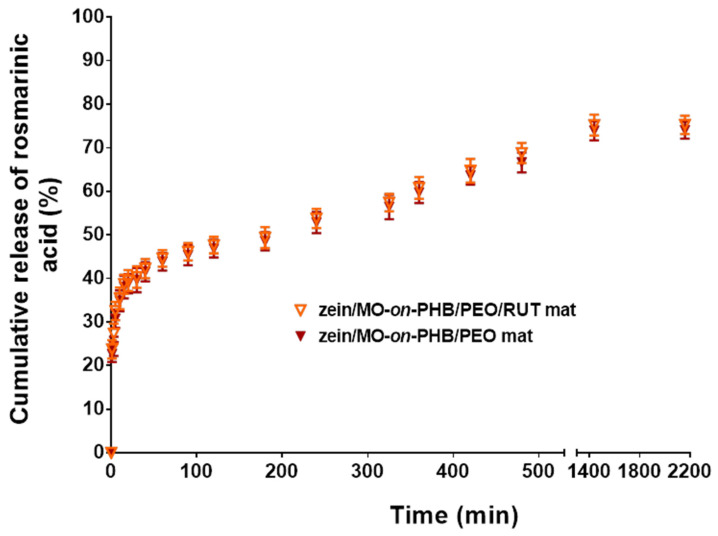
In vitro study of the release profiles of rosmarinic acid from MO loaded in the mats: zein/MO-*on*-PHB/PEO/RUT and zein/MO-*on*-PHB/PEO, in PBS/Tween 80 (99.2/0.8 *v*/*v*); pH 7.4; 37 °C.

The in vitro release behavior of rosmarinic acid, the major bioactive constituent of the MO extract, from MO-containing mats was evaluated using HPLC-DAD-ESI/MS analysis. An initial burst release, a second stage of sustained release, and the attainment of a plateau after 1440 min were also observed for the PHB/PEO or PHB/PEO/RUT mats decorated with zein/MO particles (Figure 7). The amount of rosmarinic acid released from the zein/MO-*on*-PHB/PEO/RUT and zein/MO-*on*-PHB/PEO mats during the initial 20 min was approximately 39.5% and 38.8%, respectively, while after 1440 min the released amount reached 75.2% and 73.9%, respectively. The detected sustained release of rosmarinic acid suggests that the MO-containing materials are prospective systems for achieving prolonged biological activity.

### 3.10. Antioxidant Capacity of the Fibrous Materials

Evidence from in vivo studies suggests that oxidative damage that is a critical factor in the aging process and the etiology of cancer and neurodegenerative disorders often stems more significantly from a deficiency in endogenous antioxidant defenses than from an isolated escalation in reactive oxygen species (ROS) production [64]. Consequently, there is a substantial scientific interest in developing fibrous materials containing compounds with antioxidant activity to provide cellular protection against the destructive changes induced by oxidative imbalance.

**Figure 8:**
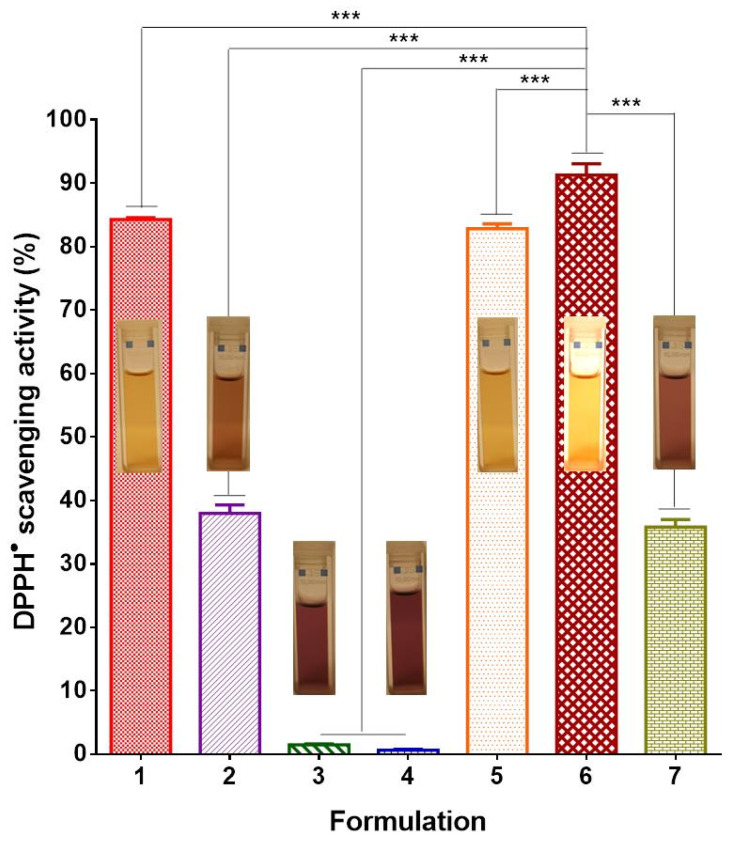
Antioxidant activity of: 1—RUT solution; 2—extract of MO solution; 3—PHB mat; 4—PHB/PEO mat; 5—PHB/PEO/RUT mat; 6—zein/MO-*on*-PHB/PEO/RUT mat; and 7—zein/MO-*on*-PHB/PEO mat. *** *p* < 0.001. Digital images of the solutions are presented.

In this study, the antioxidant capacity of RUT-loaded PHB/PEO mats, zein/MO-*on*-PHB/PEO/RUT mats and zein/MO-*on*-PHB/PEO mats was assessed using a DPPH^•^ radical scavenging assay. The antioxidant activity of the PHB and PHB/PEO mats was estimated, as well. As shown in Figure 8, PHB and PHB/PEO mats exhibited negligible antioxidant capacity, with DPPH^•^ absorbance decreasing by ca. 1.5% and 0.6%, respectively. Additionally, no visible color change in the DPPH^•^ solution was detected when it was in contact with these fibrous materials. After 30 min of exposure to RUT-loaded PHB/PEO mats, the absorbance of the DPPH^•^ solution showed a significant decrease of ca. 83%. No substantial difference was found between the DPPH^•^ radical scavenging activity of free RUT solution and that of RUT-containing PHB/PEO mats (Figure 8). Correspondingly, the DPPH^•^ solution exhibited a color change from violet to yellow upon interaction with the RUT-loaded mats. The antioxidant capacity of zein/MO-*on*-PHB/PEG mats (35.8 ± 1.2%) was comparable to that of the MO solution (37.9 ± 1.4%). Notably, PHB/PEO mats containing both RUT and MO demonstrated the highest antioxidant activity, with a reduction in DPPH^•^ absorbance up to 91.3 ± 1.8%. This value exceeds that detected for RUT-loaded PHB/PEO mats (~83%) and for PHB/PEO mats decorated with zein/MO particles (~36%). The enhanced activity is most likely due to the combined antioxidant effects of RUT [65] and extract of MO [66]. Overall, the DPPH^•^ test findings revealed that RUT and MO retained their radical scavenging capacity when incorporated into the fibers or in particles, respectively.

### 3.11. Assessment of the Cytotoxicity of the Fibrous Mats Against SH-4 and HaCaT Cells by Performing a NRU Assay

First, in vitro cytotoxicity of RUT and MO against human melanoma SH-4 cells and non-cancerous HaCaT keratinocytes was investigated using the NRU assay and the IC_50_ values, defined as the concentration needed to inhibit 50% of cell proliferation, were calculated. The IC_50_ value of free RUT in respect to SH-4 cells was determined to be 76.09 μg/mL after 24 h of incubation and 111.3 μg/mL after 48 h (Supplementary Materials, Figure S7a,b). It was found that at concentrations ranging from 3.125 μg/mL to 400 μg/mL RUT showed no cytotoxic effect on HaCaT cells (Supplementary Materials, Figure S7c,d). The IC_50_ value for MO for SH-4 cells was 297.8 μg/mL after 24 h and 142.7 μg/mL—after 48 h, for HaCaT cells the IC_50_ value was 1293 μg/mL at 24 h and decreased to 830.4 μg/mL after 48 h (Supplementary Materials, Figure S7e–h). The results indicated that RUT and MO are less cytotoxic to normal cells than to cancer cells.

**Figure 9:**
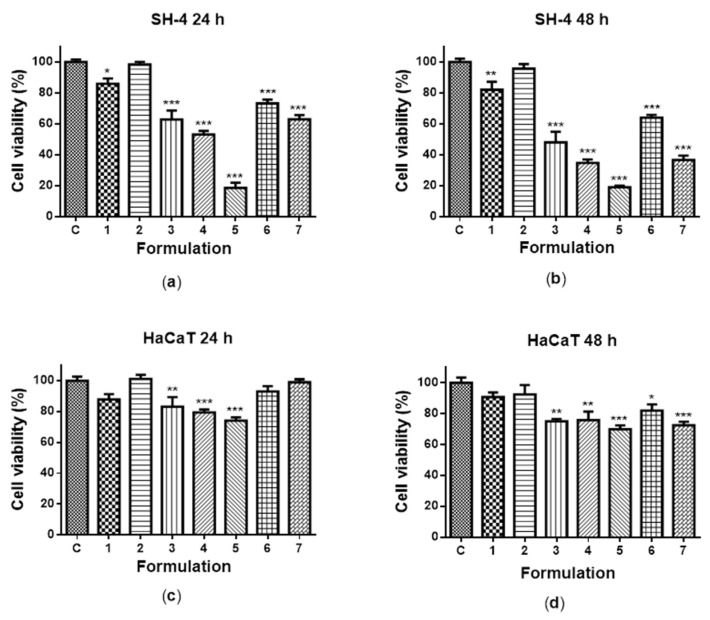
Cell viability of SH-4 melanoma or HaCaT non-cancerous cells estimated by NRU assay after 24 h (**a**,**c**) and 48 h (**b**,**d**) after contact with: C—SH-4 or HaCaT cells (control); 1—PHB mat; 2—PHB/PEO mat; 3—PBH/PEO/RUT mat; 4—zein/MO-*on*-PHB/PEO mat; 5—zein/MO-*on*-PHB/PEO/RUT mat; 6—solution of RUT; 7—solution of MO extract. All formulations containing RUT and/or MO were investigated at a concentration of RUT and MO of 300 μg/mL and 250 μg/mL, respectively. * *p* < 0.05, ** *p* < 0.01, *** *p* < 0.001.

The anticancer efficacy of the fabricated mats loaded with RUT and/or MO against SH-4 human melanoma cells was also tested by conducting a NRU assay. The cytotoxicity of the fibrous materials against non-cancerous HaCaT human keratinocytes was also estimated. The RUT and MO extract were used as positive controls and cells, cultured in medium only, as negative controls. As shown in Figure 9a, treatment with PHB mat at the 24th hour resulted in a non-significant reduction in the percentage of viable SH-4 cells compared to the untreated control. No statistically significant antiproliferative activity was detected for PHB/PEO mats. Conversely, the fibrous materials loaded with RUT or MO were effective in suppressing the proliferation of SH-4 cells. For the respective mats, cell viability declined to about 63% and 53%, respectively (Figure 9a). Among the fibrous materials, those loaded with a combination of both RUT and MO extract displayed the greatest inhibitory effect on SH-4 cells (18.7 ± 9.0% viability). The data indicate that the fibrous materials loaded with RUT and/or MO exhibited the highest antiproliferative activity following 48 h of incubation. After 48 h of treatment, the zein/MO-*on*-PHB/PEO/RUT mats caused a more pronounced reduction in SH-4 cells viability (19.1 ± 2.4%) compared to the PHB/PEO/RUT mats (48.1 ± 17.0%) and to the zein/MO-*on*-PHB/PEO mats (34.8 ± 5.4%). The free RUT (64.0 ± 4.3%) and free MO extract (36.8 ± 6.7%) possessed an inhibitory effect on the SH-4 cells viability slightly lower than that of the fibrous materials loaded with RUT and MO (Figure 9b).

Compared with SH-4 melanoma cells, normal non-cancerous HaCaT keratinocytes were less sensitive to the effects of RUT- and/or MO-loaded fibrous materials and free RUT or MO, as illustrated in Figure 9c,d. After 24 h of exposure, the viability of HaCaT cells was 83.1 ± 13.8%, 79.4 ± 4.9%, and 74.1 ± 4.2% for PHB/PEO/RUT, zein/MO-*on*-PHB/PEO and zein/MO-*on*-PHB/PEO/RUT mats, respectively (Figure 9c). Free RUT and MO had no cytotoxic effect on HaCaT cells.

Cell viability slowly declined as the exposure period increased. After 48 h of incubation, the cell viability values decreased to 75.1 ± 3.3% for PHB/PEO/RUT mats, 75.8 ± 12.5% for zein/MO-*on*-PHB/PEO mats, and 69.9 ± 4.9% for zein/MO-*on*-PHB/PEO/RUT mats (Figure 9d). Approximately 82.0% and 72.6% of HaCaT cells remained viable after contact with the free RUT and MO, respectively. The findings from the cell viability study revealed that fibrous materials loaded with RUT, MO or both markedly inhibited the viability of melanoma cancer cells, whereas their cytotoxic effect on non-cancerous cells was comparatively lower.

### 3.12. Fluorescence Microscopy Analysis for Evaluation of Apoptosis

**Figure 10:**
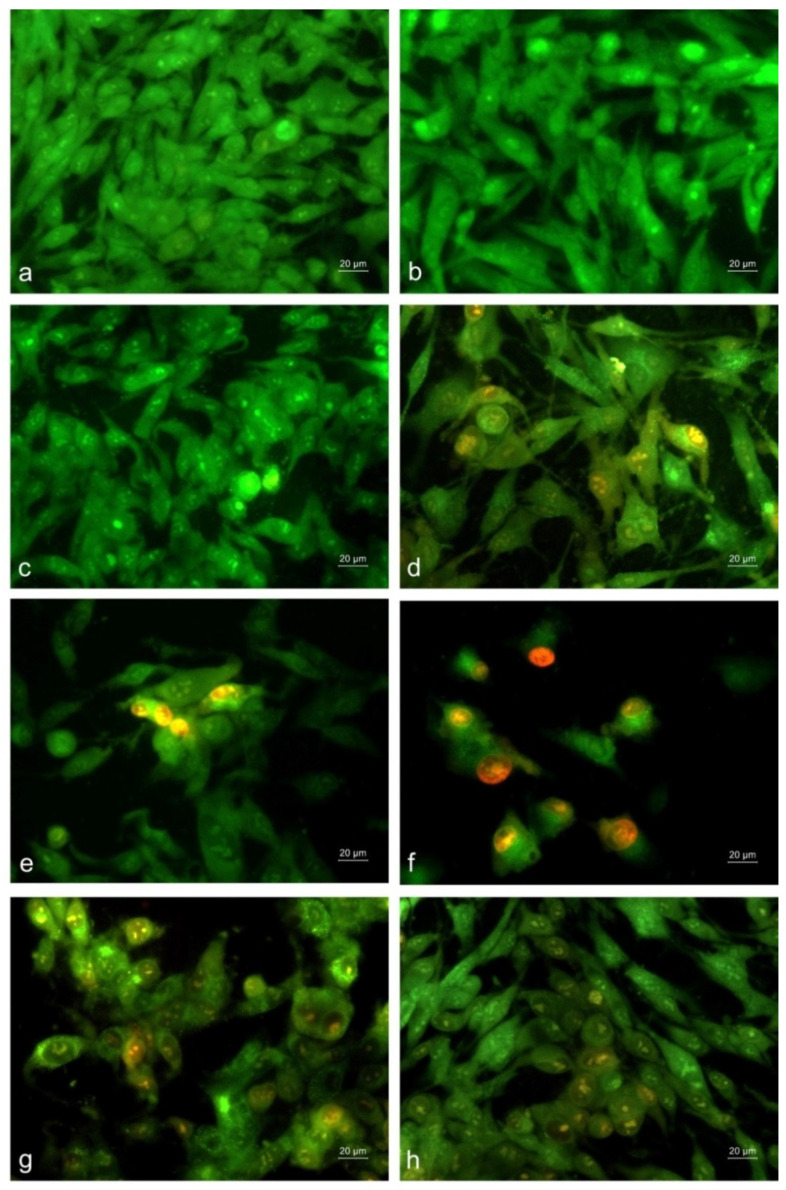
Fluorescence images of SH-4 human melanoma cells stained with AO/EtBr after 24 h of incubation with: (**a**) untreated SH-4 cells (control); (**b**) PHB mat; (**c**) PHB/PEO mat; (**d**) PHB/PEO/RUT mat; (**e**) zein/MO-*on*-PHB/PEO mat; (**f**) zein/MO-*on*-PHB/PEO/RUT mat; (**g**) solution of RUT; and (**h**) solution of MO extract. Bar = 20 μm. All formulations containing RUT and/or MO were investigated at a concentration of RUT and MO of 300 μg/mL and 250 μg/mL, respectively.

In order to clarify whether the studied fibrous materials reduced SH-4 melanoma cell viability through the induction of apoptosis, morphological changes in the cells were analyzed by fluorescence microscopy after AO/EtBr staining. As shown in Figure 10a, the untreated SH-4 melanoma cells (negative control) exhibited typical morphology, including spindle-shaped, elongated, and polygonal cells with intact membranes. These viable cells showed mainly green fluorescence. In the case of cells treated with PHB and PHB/PEO mats, comparable morphological characteristics were observed, although a slight reduction in cell density and confluency, as well as the presence of occasional rounded cells, was noted. Predominantly green fluorescent cells were visible, indicating preserved viability (Figure 10b,c). The morphological changes, such as bright yellow-orange staining of the nucleus were detected in SH-4 cells upon their incubation on PHB/PEO/RUT and zein/MO-*on*-PHB/PEO mats (Figure 10d,e). Cells exhibiting early apoptotic features, such as membrane blebbing and nuclear chromatin condensation, were detected. A decrease in cell density and monolayer confluency was also registered. The most distinct alterations in cell morphology were observed when cells were exposed to mats containing both RUT and MO, where only a limited number of viable cells remained attached and severe cellular destruction predominated. In this case, an increase in orange/red fluorescence intensity, indicating compromised membrane integrity, and nuclei with aggregated and condensed chromatin, characteristic of late-stage apoptosis, were observed (Figure 10f). The presence of rounded cells, cell shrinkage, nucleus fragmentation, blebbing of the cell membrane, and appearance of apoptotic bodies were also detected. After treatment with a solution of RUT or MO, cells displaying morphological features of early apoptosis, including green-stained cells with bright orange areas of condensed or fragmented nuclear chromatin, were observed (Figure 10g,h).

**Figure 11:**
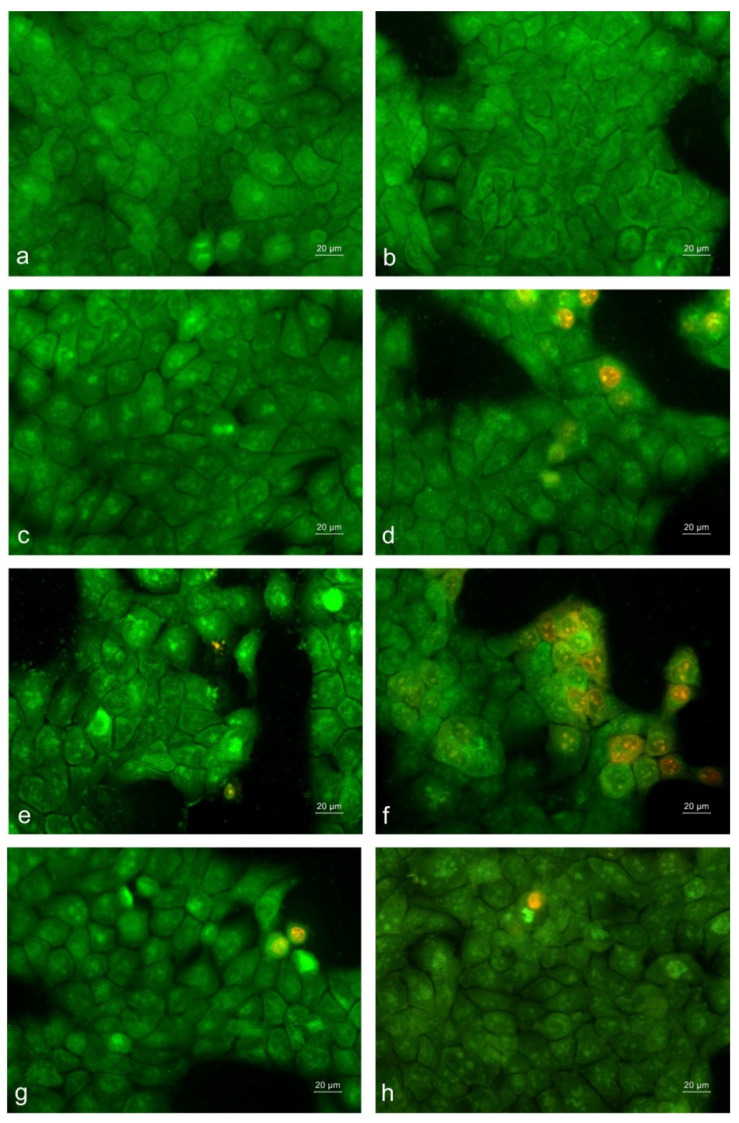
Fluorescence images of HaCaT human non-cancerous keratinocytes stained with AO/EtBr after 24 h of incubation with: (**a**) untreated HaCaT cells (control); (**b**) PHB mat; (**c**) PHB/PEO mat; (**d**) PHB/PEO/RUT mat; (**e**) zein/MO-*on*-PHB/PEO mat; (**f**) zein/MO-*on*-PHB/PEO/RUT mat; (**g**) solution of RUT; and (**h**) solution of MO extract. Bar = 20 μm. All formulations containing RUT and/or MO were investigated at a concentration of RUT and MO of 300 μg/mL and 250 μg/mL, respectively.

As seen in Figure 11d,e, non-cancerous HaCaT keratinocytes displayed only minor morphological alterations, predominantly associated with early apoptosis, after exposure to the tested mats containing RUT or MO. The strongest cytotoxic effect was detected for the zein/MO-*on*-PHB/PEO/RUT mat, where partial loss of intercellular contacts, cell shrinkage, and disrupted cellular organization were evident. Despite these alterations, a substantial proportion of the cells remained viable, indicating the greater resistance of HaCaT cells to the treatment (Figure 11f). Notably, keratinocytes incubated with PHB and PHB/PEO mats showed only minor alterations in a small number of cells, while largely maintaining morphology comparable to that of the untreated control cells (Figure 11a–c). The results showed that the cytotoxic effect remained significantly weaker than that observed in SH-4 melanoma cells, suggesting the selectivity of the fibrous mats toward cancer cells.

To further assess damage in the nuclei of SH-4 cells, DAPI staining was performed. Untreated cells exhibited normal oval nuclei that were uniformly stained, with a homogeneous chromatin structure and no signs of apoptosis (Supplementary Materials, Figure S8a). After treatment with PHB and PHB/PEO mats, nuclear morphology remained relatively well preserved, although some cells exhibited increased fluorescence intensity and early chromatin condensation, indicative of initial apoptotic changes (Supplementary Materials, Figure S8b,c).

More substantial nuclear damage was evident when cells were exposed to PHB/PEO/RUT and zein/MO-*on*-PHB/PEO mats, where many nuclei displayed marked chromatin condensation, nuclear fragmentation, and brightly fluorescent apoptotic bodies (Supplementary Materials, Figure S8d,e). The strongest nuclear alterations were observed in cells that were in contact with zein/MO-*on*-PHB/PEO/RUT mats, with a large proportion of nuclei showing intense fluorescence, highly condensed chromatin, and extensive fragmentation, characteristic of late-stage apoptosis (Supplementary Materials, Figure S8f). Different nuclear abnormalities were also observed after treatment with free RUT and MO solutions. Some nuclei appeared irregular in shape and unevenly stained, with moderate to strong chromatin condensation and partial nuclear fragmentation, further confirming the induction of apoptotic cell death (Supplementary Materials, Figure S8g,h).

The results obtained from fluorescence microscopy analyses of untreated and treated non-cancerous HaCaT keratinocytes stained with DAPI are shown in Figure S9 (Supplementary Materials). DAPI staining of control HaCaT cells revealed normal round to oval-shaped nuclei with homogeneous chromatin distribution and no apoptotic features (Supplementary Materials, Figure S9a). PHB and PHB/PEO mats induced only minimal early apoptotic changes in the nuclear morphology, affecting only a small proportion of the cells (Supplementary Materials, Figure S9b,c). Exposure to PHB/PEO/RUT and zein/MO-*on*-PHB/PEO mats resulted predominantly in moderate apoptotic alterations, with relatively preserved nuclear morphology and only occasional fragmented nuclei detected (Supplementary Materials, Figure S9d,e). The most pronounced nuclear damage and apoptotic body formation were detected in cells incubated with zein/MO-*on*-PHB/PEO/RUT mats (Supplementary Materials, Figure S9f). Free RUT and MO solutions caused only mild nuclear alterations, with most nuclei remaining relatively intact (Supplementary Materials, Figure S9g,h).

The fluorescence microscopy analysis was conducted for qualitative assessment of the morphological changes in the SH-4 melanoma cells and non-cancerous HaCaT cells. This analysis revealed that the mats containing RUT and/or MO exhibited greater cytotoxic effects against SH-4 melanoma cancer cells than against HaCaT cells, which was consistent with the findings of the cell viability assay. For SH-4 cells, mats loaded with RUT or MO, and especially those loaded with both RUT and MO, induced pronounced apoptotic changes, including intense chromatin condensation, strong nuclear fluorescence, extensive nuclear fragmentation, and apoptotic body formation, characteristic of late-stage apoptosis. In contrast, HaCaT cells exhibited mainly moderate apoptotic alterations under the same treatment conditions, with relatively preserved nuclear morphology and only occasional nuclear fragmentation observed. The pronounced morphological changes detected in melanoma cancer cells following treatment with the prepared fibrous mats loaded with RUT and/or MO indicated that these materials induced programmed cell death through apoptosis. Further quantitative assessment of apoptosis should be performed to elucidate the molecular pathways responsible for the anticancer activities of the fabricated fibrous materials. Considering the potential clinical applications of the newly developed drug-loaded mats, subsequent in vivo studies are required to validate their efficacy and safety.

## 4. Conclusions

Composite fibrous materials with spatially separated incorporation of rutin (RUT) and *Melissa officinalis* extract (MO) were successfully fabricated through simultaneous electrospinning of PHB/PEO/RUT solutions and electrospraying of zein/MO dispersions. The designed architecture enabled compartmentalization of the two bioactive agents while preserving them in an amorphous state. The incorporation of PEO imparted high hydrophilicity to the fibrous matrices and facilitated rapid RUT release, whereas MO entrapped within zein particles exhibited a more sustained release profile. The dual-loaded materials displayed enhanced antioxidant activity compared with systems containing only one bioactive component. Importantly, the composite mats showed preferential antiproliferative activity toward SH-4 melanoma cells while exhibiting substantially lower toxicity toward non-cancerous HaCaT keratinocytes, indicating a degree of biological selectivity. Overall, the results demonstrate that spatially engineered electrospun/electrosprayed PHB/PEO/zein systems represent promising multifunctional platforms for localized delivery of natural bioactive compounds with antioxidant and anticancer potential.

## Data Availability

The original contributions presented in this study are included in the article/Supplementary Materials. Further inquiries can be directed to the corresponding author.

## Supplementary Materials

The following supporting information can be downloaded at https://www.mdpi.com/article/10.3390/polym18141774/s1, Scheme S1: Schematic representation of fibers from PHB (a) and PHB/PEO (b) prepared by electrospinning; Figure S1: SEM micrograph and fiber diameter distribution of the electrospun PHB mat; Figure S2: ATR-FTIR spectra of: (a) PHB mat, (b) PEO powder, (c) zein powder, (d) RUT and (e) MO; Figure S3: TGA thermograms of PHB mat, PEO powder, zein powder, PHB/PEO mat, zein/MO-*on*-PHB/PEO mat, PHB/PEO/RUT mat and zein/MO-*on*-PHB/PEO/RUT mat; Figure S4: TGA thermograms of MO and RUT powder; Figure S5: DSC thermograms of PHB mat (a); PHB/PEO mat (b); PHB/PEO/RUT mat (c); zein/MO-*on*-PHB/PEO mat (d), zein/MO-*on*-PHB/PEO/RUT mat (e); and PEO powder (f); Figure S6: DSC thermograms of rutin (A) and zein (B); Figure S7: Effect of free RUT (a–d) and free MO (e–h) on the viability of the SH-4 melanoma cells (a,b,e,f) or HaCaT non-cancerous keratinocytes (c,d,g,h) incubated 24 h and 48 h in the presence of RUT or MO. The RUT concentration was from 3.125 to 400 μg/mL. The MO concentration was from 15.625 to 2000 μg/mL Data are means ± SD of six replicates. *** *p* < 0.001, ** *p* < 0.01; Figure S8: Fluorescence images of SH-4 human melanoma cells stained with DAPI after 24 h of incubation with: (a) untreated SH-4 cells (control); (b) PHB mat; (c) PHB/PEO mat; (d) PHB/PEO/RUT mat; (e) zein/MO-*on*-PHB/PEO mat; (f) zein/MO-*on*-PHB/PEO/RUT mat; (g) solution of RUT; and (h) solution of MO extract. Bar = 20 μm. All formulations containing RUT and/or MO were investigated at a concentration of RUT and MO of 300 μg/mL and 250 μg/mL, respectively; Figure S9: Fluorescence images of HaCaT human non-cancerous keratinocytes stained with DAPI after 24 h of incubation with: (a) untreated HaCaT cells (control); (b) PHB mat; (c) PHB/PEO mat; (d) PHB/PEO/RUT mat; (e) zein/MO-*on*-PHB/PEO mat; (f) zein/MO-*on*-PHB/PEO/RUT mat; (g) solution of RUT; and (h) solution of MO extract. Bar = 20 μm. All formulations containing RUT and/or MO were investigated at a concentration of RUT and MO of 300 μg/mL and 250 μg/mL, respectively; Table S1: Dynamic viscosity (η) and conductivity (σ) of the spinning solutions, and average fiber diameter of the electrospun mats; Table S2: Tensile characteristics of the fibrous materials.
