# Supplementary material for: Spatially Compartmentalized Electrospun/Electrosprayed PHB/PEO/Zein Fibrous Platforms for Dual Delivery of Rutin and Melissa officinalis Extract
Source: Polymers (Basel). 2026 Jul 20;18(14):1774. doi: 10.3390/polym18141774 (PMC13431348; doi:10.3390/polym18141774)
Supplement: Supplementary file 1 [file polymers-18-01774-s001.zip › polymers-4436569-supplementary.pdf]

Supplementary Materials

Spatially Compartmentalized Electrospun/Electrosprayed PHB/PEO/Zein Fibrous Platforms for Dual Delivery of Rutin and *Melissa officinalis* Extract

Dilyana Paneva [1], Selin Kyuchyuk [1], Milena Ignatova [1,2,*], Nevena Manolova [1], Iliya Rashkov [1,†], Ani Georgieva [3], Reneta Toshkova [3] and Mariana Kamenova-Nacheva [4,5]

[1] Laboratory of Bioactive Polymers, Institute of Polymers, Bulgarian Academy of Sciences, Akad. G. Bonchev St, bl. 103A, 1113 Sofia, Bulgaria; panevad@polymer.bas.bg (D.P.); selin.erdinch@polymer.bas.bg (S.K.); manolova@polymer.bas.bg (N.M.); rashkov@polymer.bas.bg (I.R.)
[2] Centre of Competence "Sustainable Utilization of Bio-Resources and Waste of Medicinal and Aromatic Plants for Innovative Bioactive Products" (BIORESOURCES BG), 1000 Sofia, Bulgaria
[3] Institute of Experimental Morphology, Pathology and Anthropology with Museum, Bulgarian Academy of Sciences, Akad. G. Bonchev St, bl. 25, 1113 Sofia, Bulgaria; georgieva_any@abv.bg (A.G.); rtoshkova@bas.bg (R.T.)
[4] Institute of Organic Chemistry with Centre of Phytochemistry, Bulgarian Academy of Sciences, Akad. G. Bonchev St., bl. 9, 1113 Sofia, Bulgaria; mariana.nacheva@orgchm.bas.bg
[5] Research and Development and Innovation Consortium, Sofia Tech Park JSC, 111 Tsarigradsko Shose Blvd., 1784 Sofia, Bulgaria
* Correspondence: ignatova@polymer.bas.bg; Tel.: +359-(0)2-9793289
† This paper is dedicated to the memory of Iliya Rashkov, who passed away on 8 October 2025.

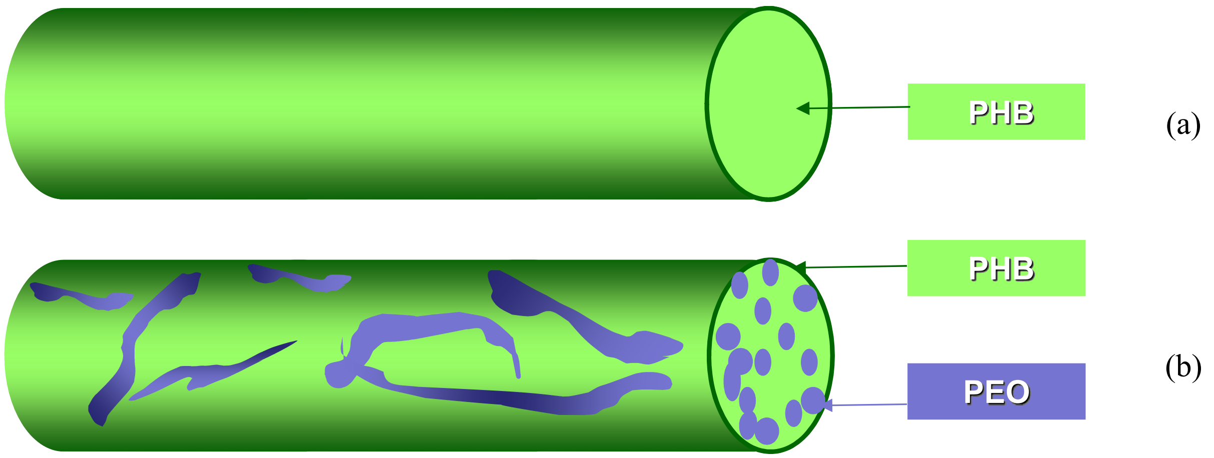

Scheme S1. Schematic representation of fibers from PHB (a) and PHB/PEO (b) prepared by electrospinning.

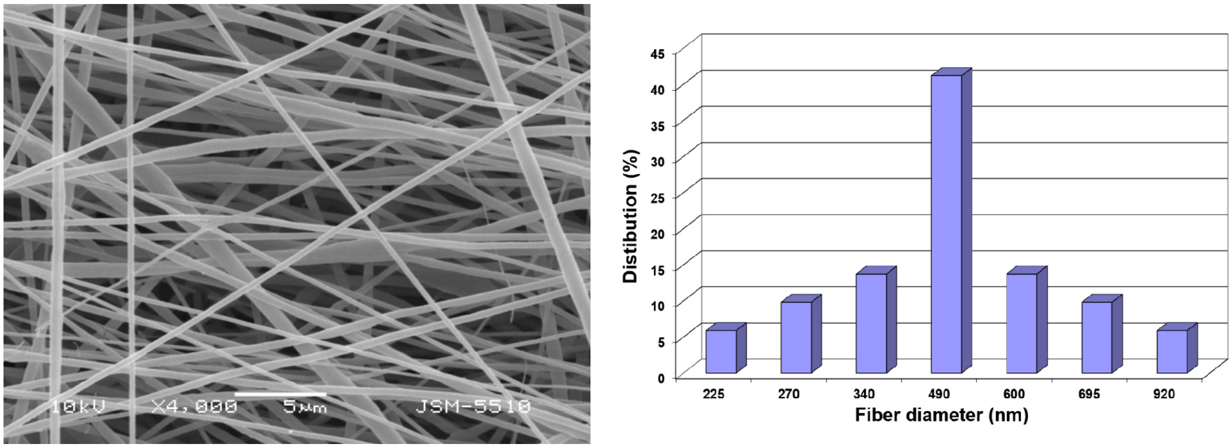

Figure S1. SEM micrograph and fiber diameter distribution of the electrospun PHB mat.

Table S1. Dynamic viscosity (η) and conductivity (σ) of the spinning solutions, and average fiber diameter of the electrospun mats.

**Electrospun mats**	**η (cP)**	**σ (μS/cm)**	**d (nm)**
PHB	670	24	495 ± 180
PHB/PEO	440	24	380 ± 140
PHB/PEO/RUT	400	24	370 ± 145

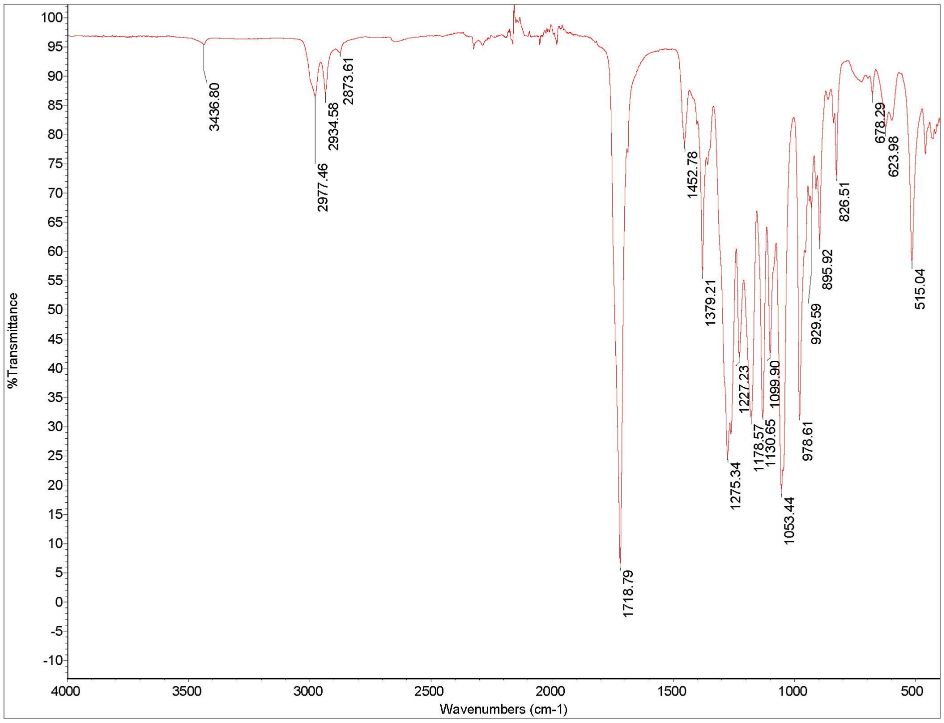
%Transmittance
Wavenumbers (cm-1)
3436.80
2977.46
2934.58
2873.61
1718.79
1452.78
1379.21
1275.34
1227.23
1178.57
1130.65
1099.90
1053.44
978.61
929.59
895.92
826.51
678.29
623.98
515.04

a

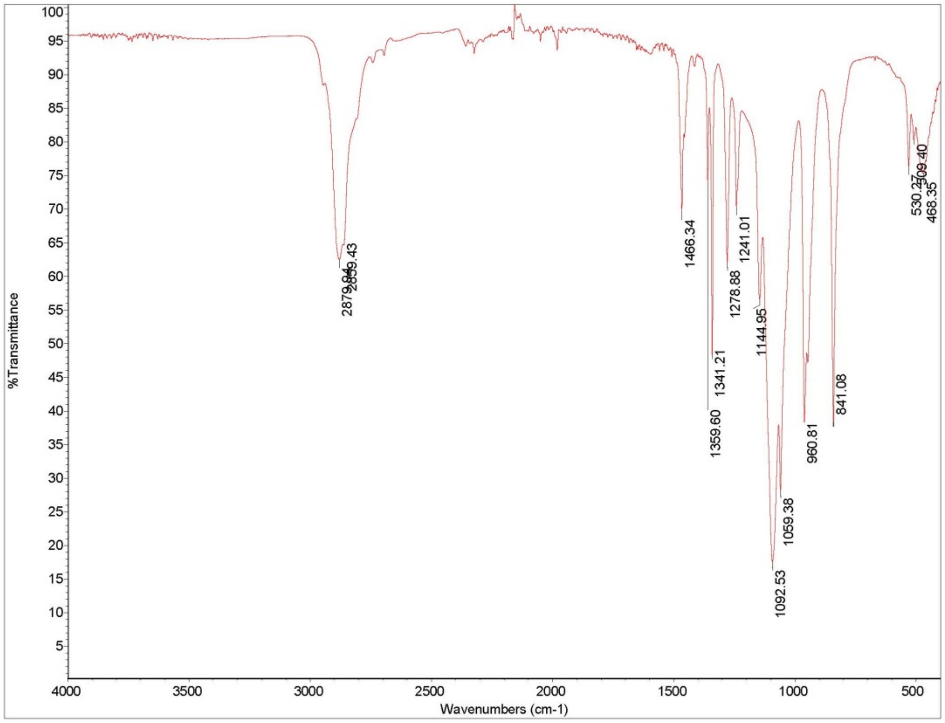
%Transmittance
Wavenumbers (cm-1)
2879.94
2859.43
1466.34
1359.60
1341.21
1278.88
1241.01
1144.95
1092.53
1059.38
960.81
841.08
530.27
509.40
468.35

b

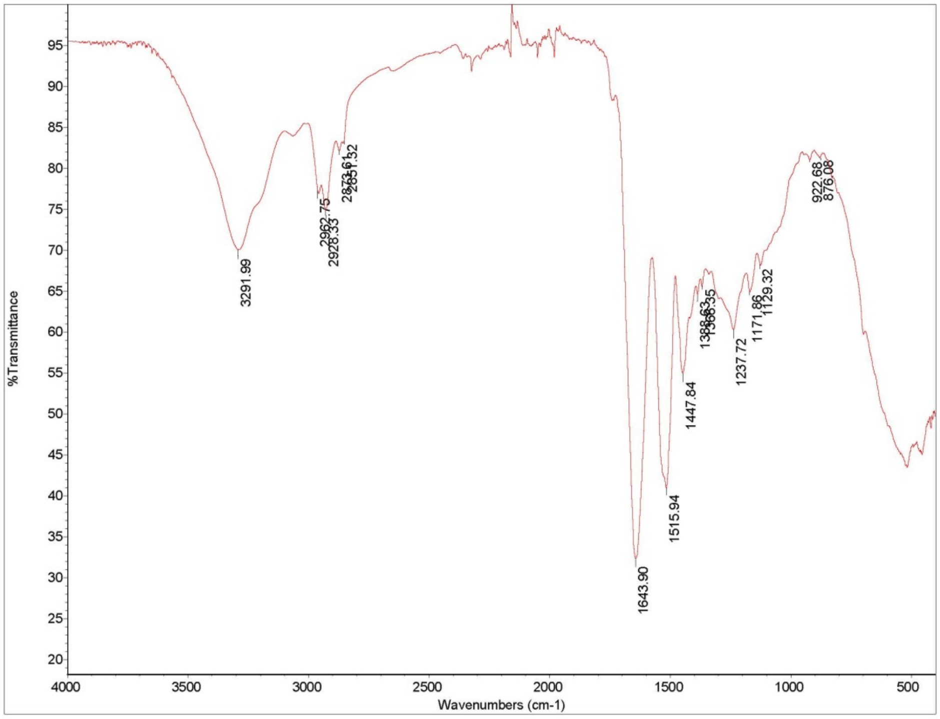
%Transmittance
Wavenumbers (cm-1)
3291.99
2962.75
2928.33
1643.90
1515.94
1447.84
1237.72
1171.86
1129.32
922.68
876.08

c

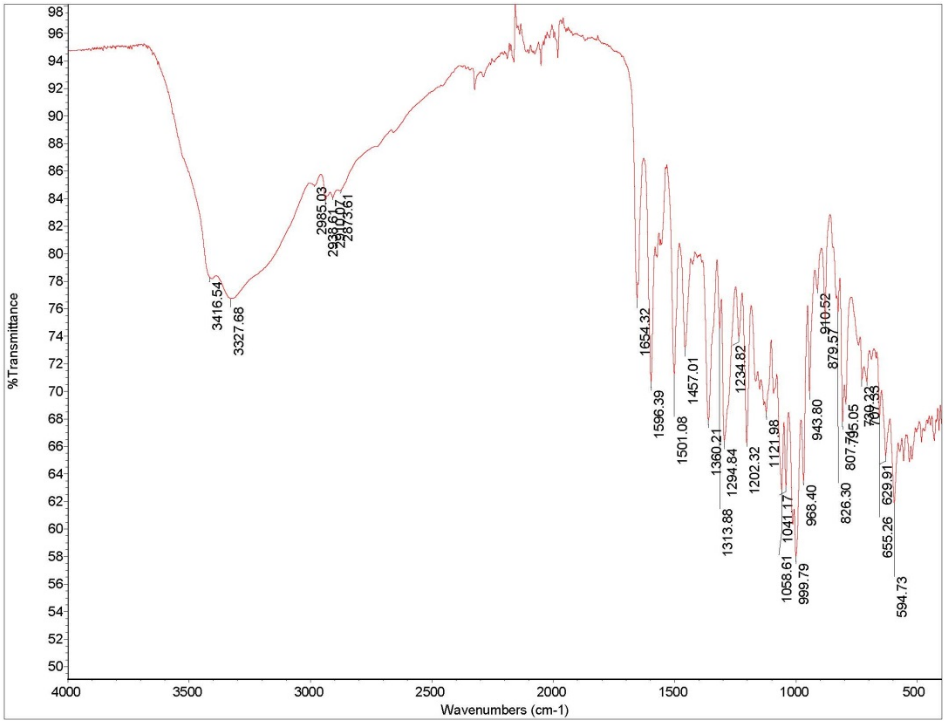
%Transmittance
Wavenumbers (cm-1)
3416.54
3327.68
2985.03
2873.61
1654.32
1596.39
1501.08
1457.01
1360.21
1313.88
1294.84
1234.82
1202.32
1121.98
1058.61
1041.17
999.79
968.40
943.80
910.52
879.57
826.30
655.26
629.91
594.73

d

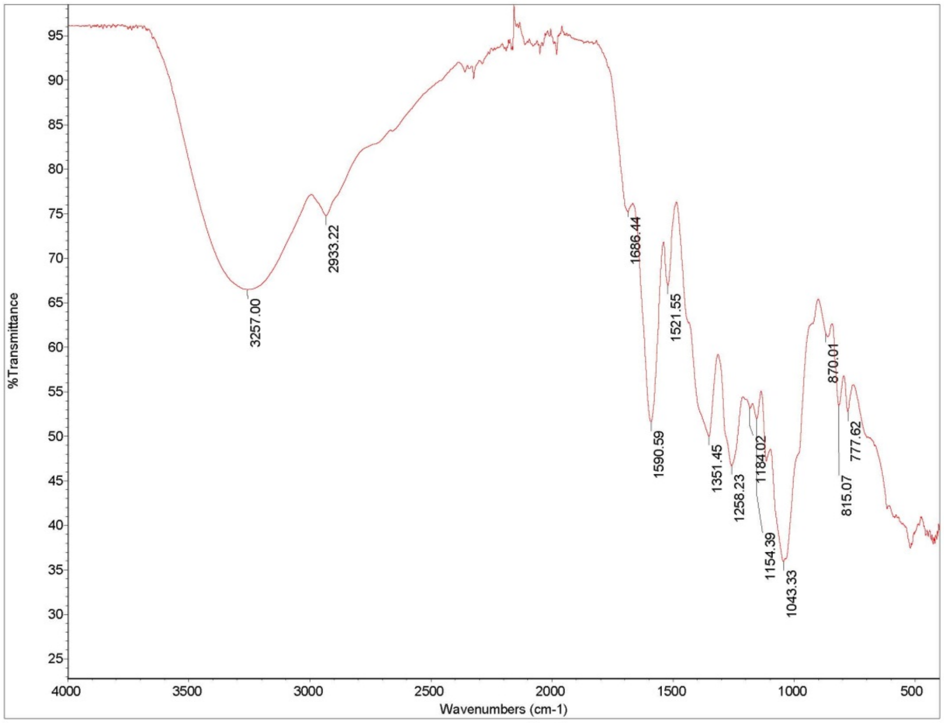

e

Figure S2. ATR-FTIR spectra of: (a) PHB mat, (b) PEO powder, (c) zein powder, (d) RUT and (e) MO.

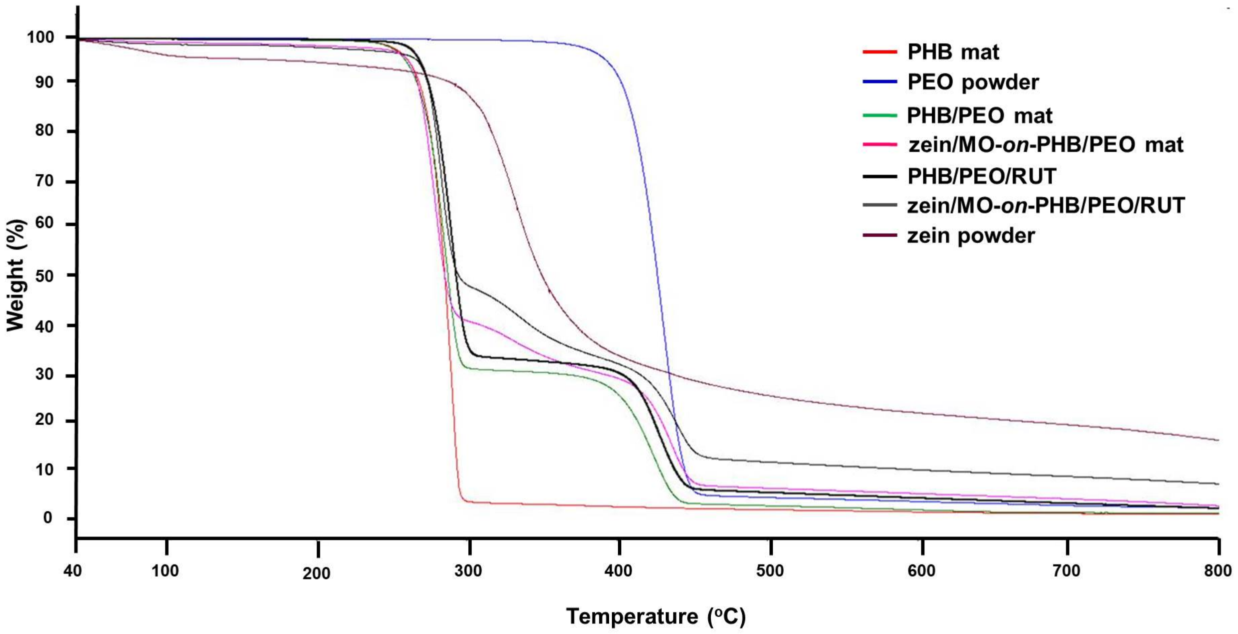

Figure S3. TGA thermograms of PHB mat, PEO powder, zein powder, PHB/PEO mat, zein/MO-*on*-PHB/PEO mat, PHB/PEO/RUT mat and zein/MO-*on*-PHB/PEO/RUT mat.

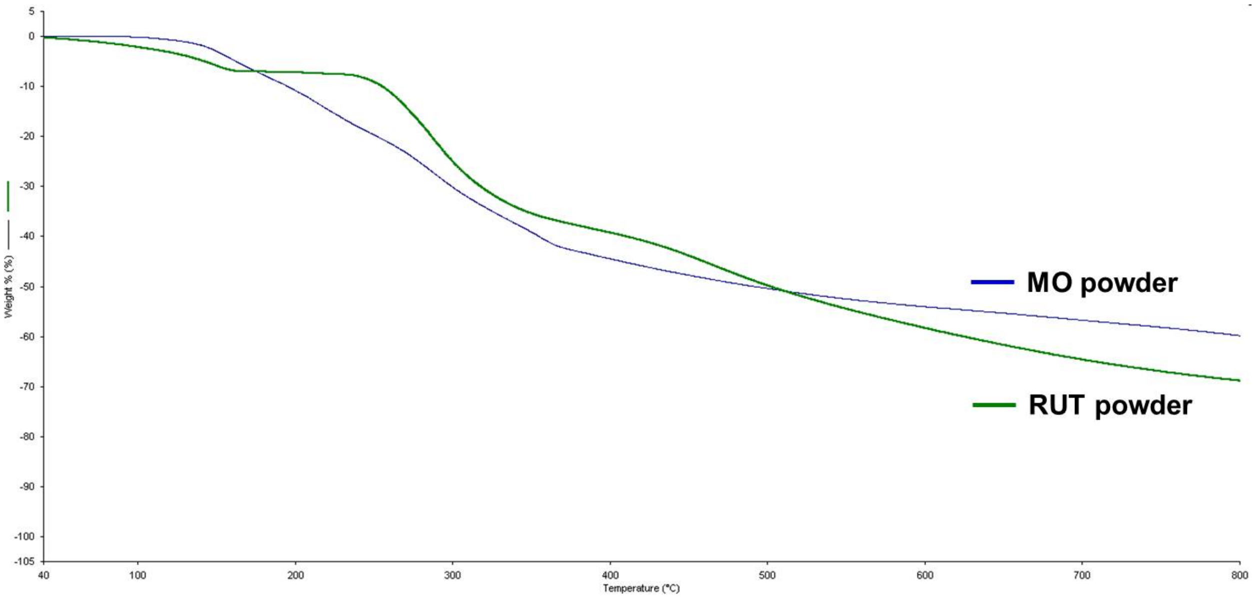

Figure S4. TGA thermograms of MO and RUT powder.

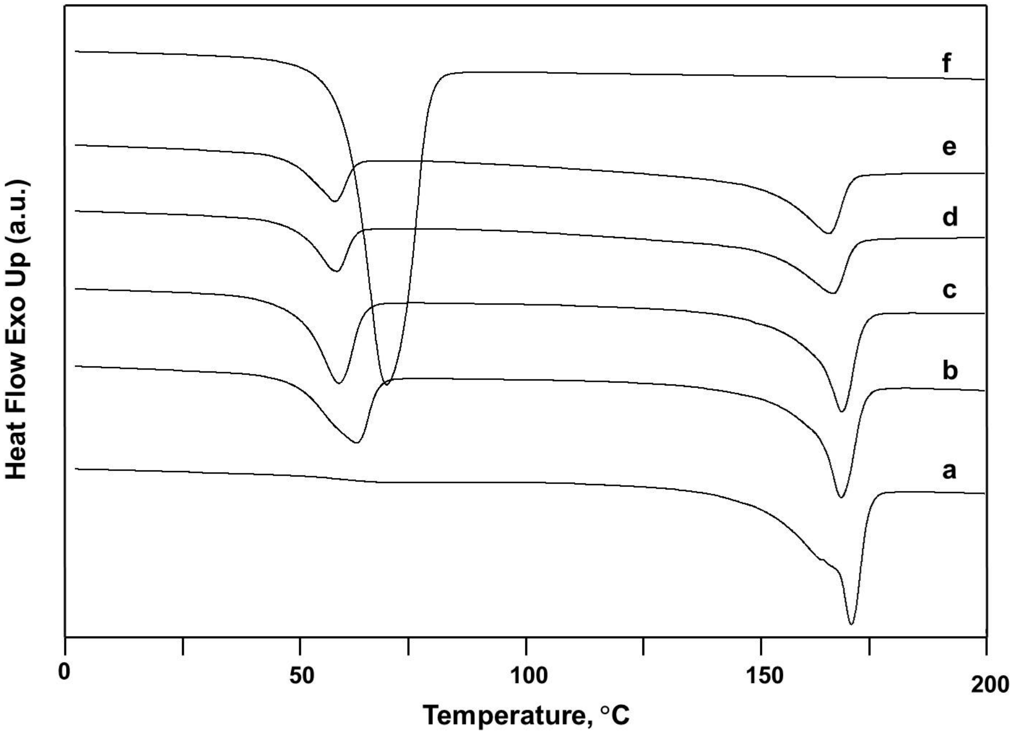

Figure S5. DSC thermograms of PHB mat (a); PHB/PEO mat (b); PHB/PEO/RUT mat (c); zein/MO-*on*-PHB/PEO mat (d), zein/MO-*on*-PHB/PEO/RUT mat (e); and PEO powder (f).

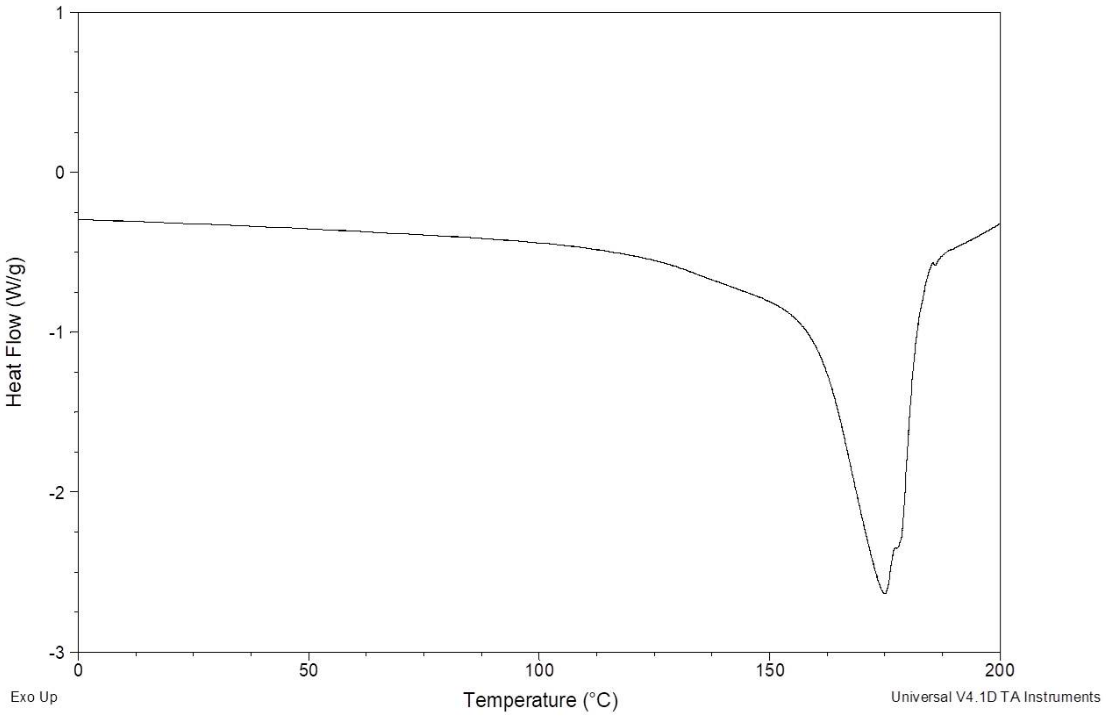

A

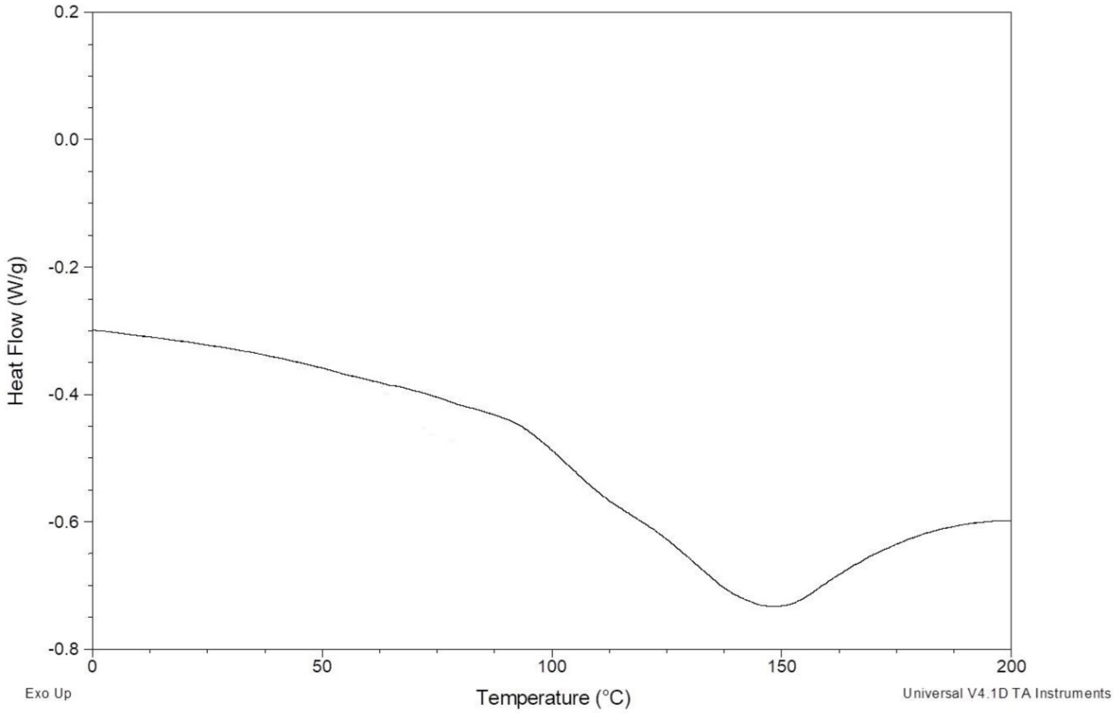

B

Figure S6. DSC thermograms of rutin (A) and zein (B).

Table S2. Tensile characteristics of the fibrous materials

Fibrous Materials	Tensile strength at Maximum Load [MPa]	Young's modulus [MPa]	Elongation at Break, %
PHB	2.45 ± 0.11	183.20± 3.10	6.00 ± 0.20
PHB/PEO	1.57 ± 0.07	39.30 ± 0.50	6.50 ± 0.15
PHB/PEO/RUT	2.20 ± 0.12	157.80 ± 2.20	3.90 ± 0.24
zein/MO-*on*-PHB/PEO	1.46 ± 0.07	83.60 ± 0.70	4.20 ± 0.20
zein/MO-*on*-PHB/PEO/RUT	1.90 ± 0.06	170.90 ± 2.30	2.10 ± 0.18

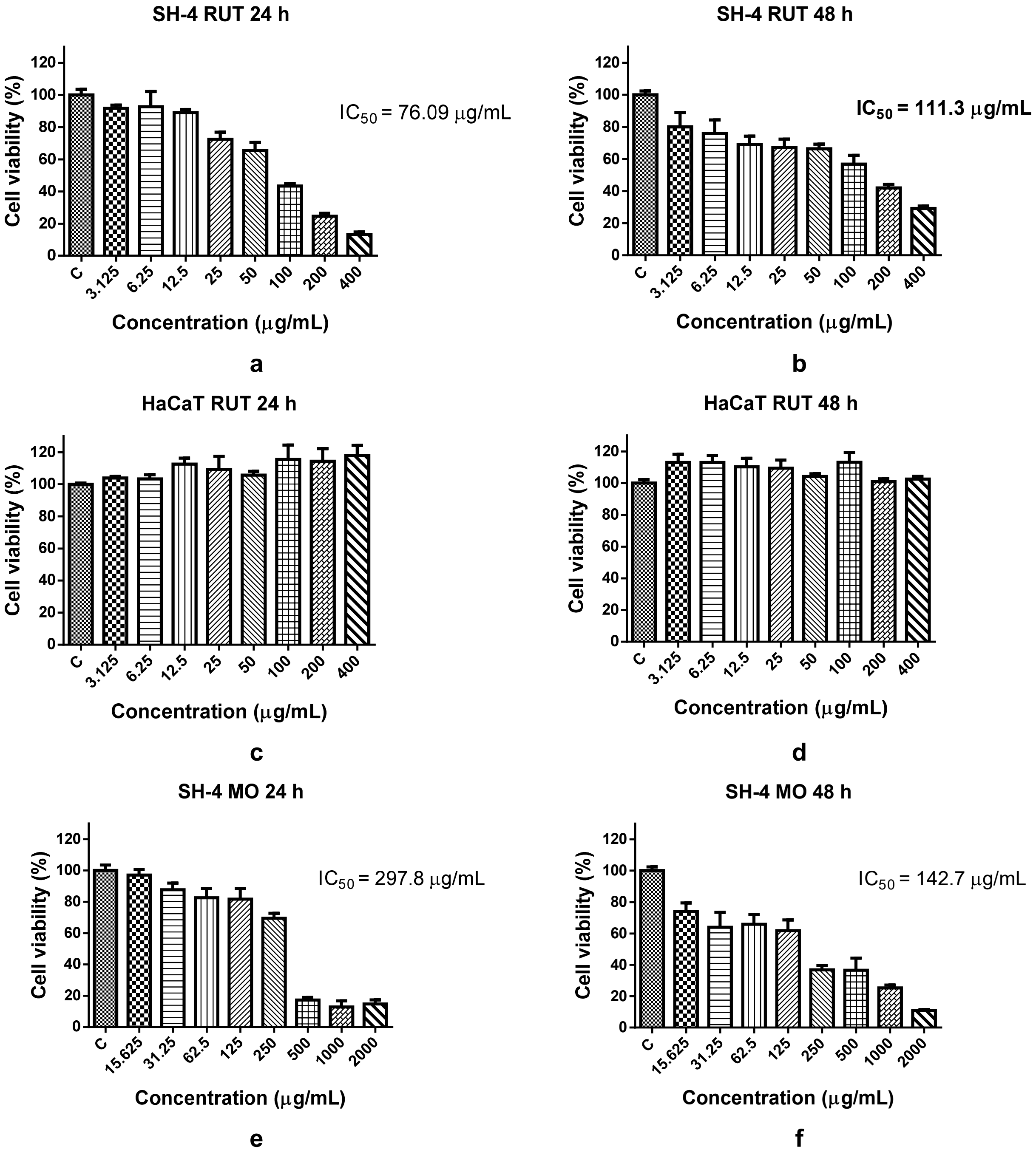
SH-4 RUT 24 h
Cell viability (%)
IC50 = 76.09 μg/mL
C
3.125
6.25
12.5
25
50
100
200
400
Concentration (μg/mL)
a
SH-4 RUT 48 h
Cell viability (%)
IC50 = 111.3 μg/mL
Concentration (μg/mL)
b
HaCaT RUT 24 h
Cell viability (%)
Concentration (μg/mL)
c
HaCaT RUT 48 h
Cell viability (%)
Concentration (μg/mL)
d
SH-4 MO 24 h
Cell viability (%)
IC50 = 297.8 μg/mL
C
15.625
31.25
62.5
125
250
500
1000
2000
Concentration (μg/mL)
e
SH-4 MO 48 h
Cell viability (%)
IC50 = 142.7 μg/mL
Concentration (μg/mL)
f

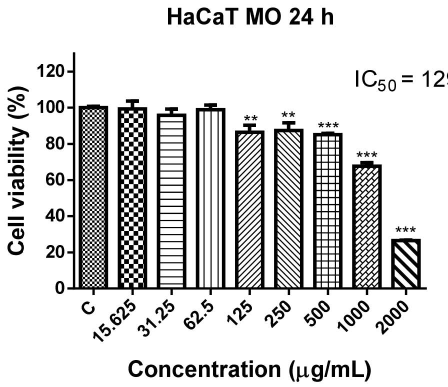

g

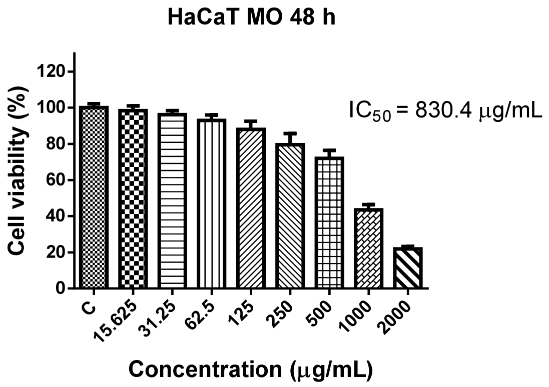

h

Figure S7. Effect of free RUT (a-d) and free MO (e-h) on the viability of the SH-4 melanoma cells (a,b,e,f) or HaCaT non-cancerous keratinocytes (c,d,g,h) incubated 24 h and 48 h in the presence of RUT or MO. The RUT concentration was from 3.125 to 400 µg/mL. The MO concentration was from 15.625 to 2000 µg/mL Data are means ± SD of six replicates. ***$p < 0.001$,**$p < 0.01$.

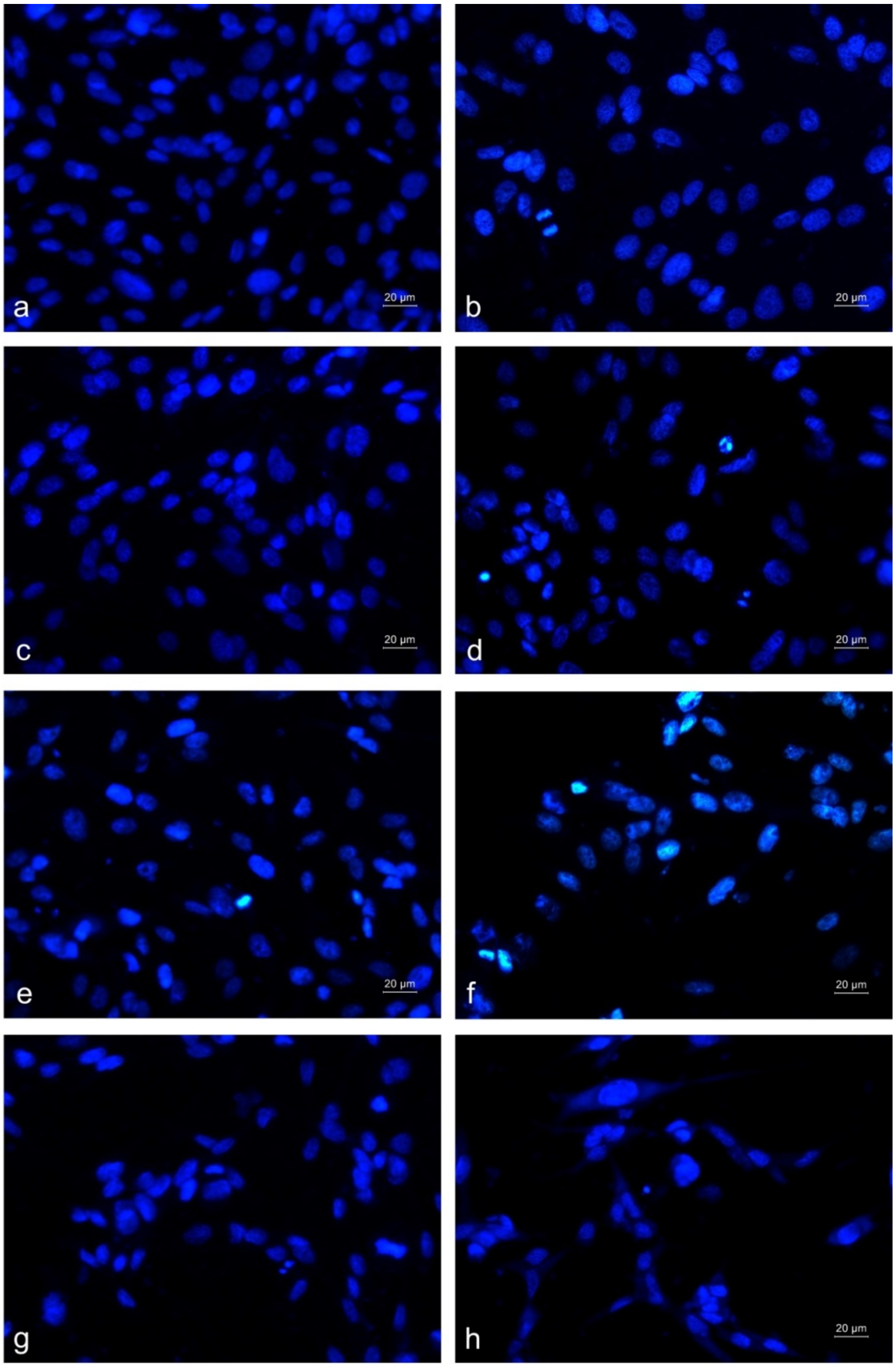

Figure S8. Fluorescence images of SH-4 human melanoma cells stained with DAPI after 24 h of incubation with: (a) untreated SH-4 cells (control); (b) PHB mat; (c) PHB/PEO mat; (d) PHB/PEO/RUT mat; (e) zein/MO-*on*-PHB/PEO mat; (f) zein/MO-*on*-PHB/PEO/RUT mat; (g) solution of RUT; and (h) solution of MO extract. Bar = 20 µm. All formulations containing RUT and/or MO were investigated at a concentration of RUT and MO of 300 µg/mL and 250 µg/mL, respectively.

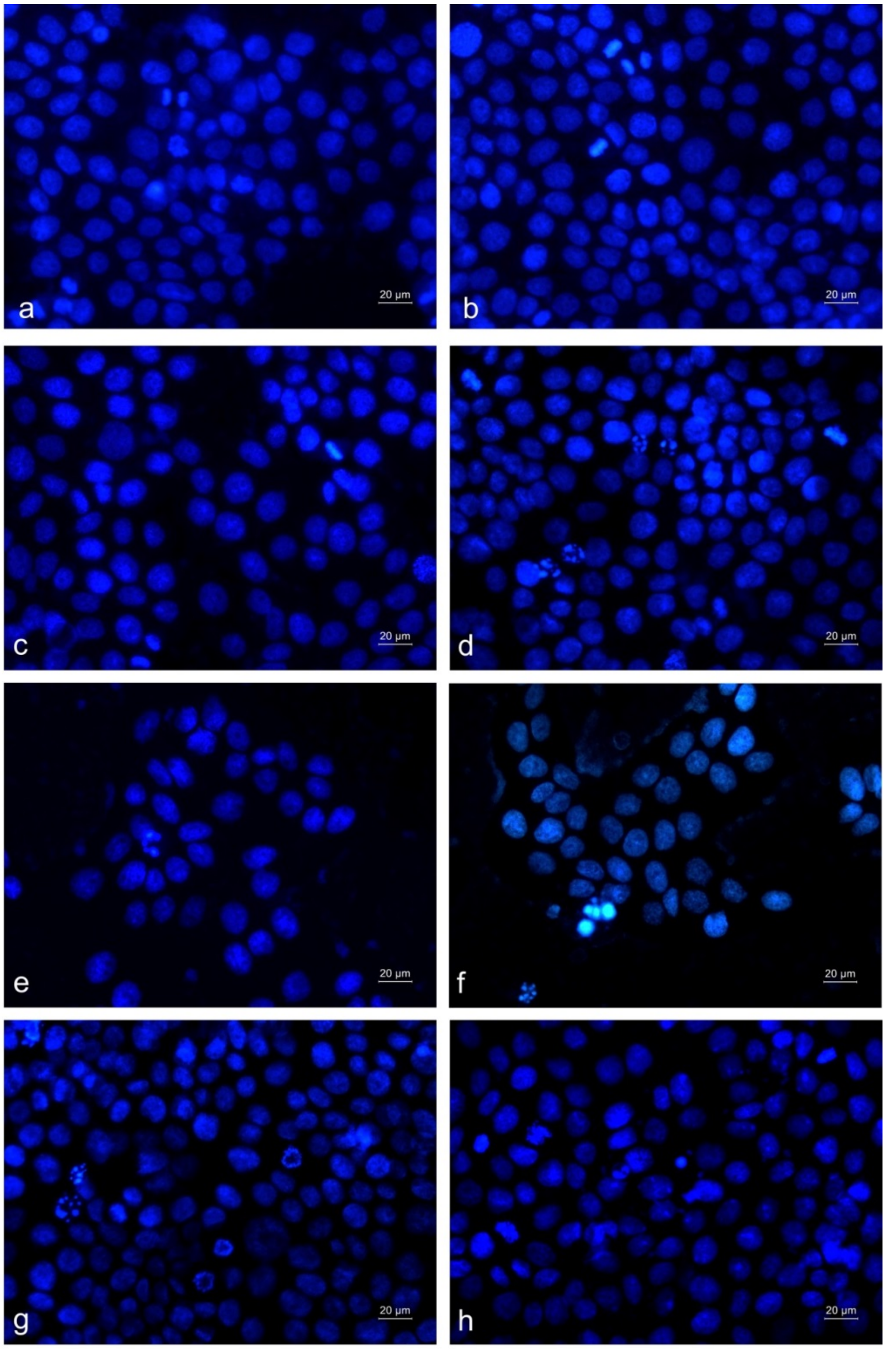

Figure S9. Fluorescence images of HaCaT human non-cancerous keratinocytes stained with DAPI after 24 h of incubation with: (a) untreated HaCaT cells (control); (b) PHB mat; (c) PHB/PEO mat; (d) PHB/PEO/RUT mat; (e) zein/MO-*on*-PHB/PEO mat; (f) zein/MO-*on*-PHB/PEO/RUT mat; (g) solution of RUT; and (h) solution of MO extract. Bar = 20 µm. All formulations containing RUT and/or MO were investigated at a concentration of RUT and MO of 300 µg/mL and 250 µg/mL, respectively.
